# Effects of the Rhizosphere Fungus *Cunninghamella bertholletiae* on the *Solanum lycopersicum* Response to Diverse Abiotic Stresses

**DOI:** 10.3390/ijms23168909

**Published:** 2022-08-10

**Authors:** Elham Ahmed Kazerooni, Sajeewa S. N. Maharachchikumbura, Abdullah Mohammed Al-Sadi, Umer Rashid, Il-Doo Kim, Sang-Mo Kang, In-Jung Lee

**Affiliations:** 1 Department of Applied Biosciences, Kyungpook National University, Daegu 41566, Korea; 2 School of Life Science and Technology, Center for Informational Biology, University of Electronic Science and Technology of China, Chengdu 611731, China; 3 Department of Plant Sciences, College of Agricultural and Marine Sciences, Sultan Qaboos University, P.O. Box 34, Al-Khoudh 123, Oman; 4 Institute of Nanoscience and Nanotechnology (ION2), Universiti Putra Malaysia, Serdang 43400, Selangor, Malaysia

**Keywords:** tomato, salinity, heavy metal, drought, antioxidant enzymes, sugar, phytohormone

## Abstract

This study examined the efficiency of fungal strain (*Cunninghamella bertholletiae*) isolated from the rhizosphere of *Solanum lycopersicum* to reduce symptoms of salinity, drought and heavy metal stresses in tomato plants. In vitro evaluation of *C. bertholletiae* demonstrated its ability to produce indole-3-Acetic Acid (IAA), ammonia and tolerate varied abiotic stresses on solid media. Tomato plants at 33 days’ old, inoculated with or without *C. bertholletiae*, were treated with 1.5% sodium chloride, 25% polyethylene glycol, 3 mM cadmium and 3 mM lead for 10 days, and the impact of *C*. *bertholletiae* on plant performance was investigated. Inoculation with *C*. *bertholletiae* enhanced plant biomass and growth attributes in stressed plants. In addition, *C*. *bertholletiae* modulated the physiochemical apparatus of stressed plants by raising chlorophyll, carotenoid, glucose, fructose, and sucrose contents, and reducing hydrogen peroxide, protein, lipid metabolism, amino acid, antioxidant activities, and abscisic acid. Gene expression analysis showed enhanced expression of *SlCDF3* and *SlICS* genes and reduced expression of *SlACCase*, *SlAOS*, *SlGRAS6*, *SlRBOHD*, *SlRING1*, *SlTAF1*, and *SlZH13* genes following *C. bertholletiae* application. In conclusion, our study supports the potential of *C*. *bertholletiae* as a biofertilizer to reduce plant damage, improve crop endurance and remediation under stress conditions.

## 1. Introduction

Plants have grown under changeable agricultural ecosystems since their emergence. Therefore, it is crucial for them to be able to respond to the varied conditions. Abiotic stresses in the shape of heat, salinity, chill, flood, water deficit, and heavy metals have a devastating impact on plant growth and yield [1,2,3,4,5]. Salinity is one of the most devastating environmental factors, magnified by the intensive salinization of lands [6]. Variable intensities of salt cause photosynthetic apparatus damage, nutritional insufficiency, oxidative injury, osmotic pressure, and ion toxicity [7,8], which interferes with all growth stages, comprising sprouting of seeds, seedling formation and fertility ratio [9]. It has been expected that water deficit frequency and severity will rise in numerous districts in the future due to global climate change [10,11]. Some consider drought as the most severe threat to global food security, and its effect has been increased owing to the reduction of water resources [12]. On top of that, soil polluted with heavy metals has become prevalent in various terrestrial ecosystems. It has been enhanced by industrial metal products, mining, municipal waste disposal, fertilizers and pesticides in agricultural systems [13]. These activities initiate the distribution of heavy metals, which might lead to food insecurity and affect ecosystem health [14]. Heavy metal accumulation causes growth reduction by changing physiological and molecular functions in plants [15].

The rhizosphere, consisting of soil around plants roots, is a native territory of thousands of beneficial microorganisms and is considered a complicated ecosystem [16,17]. Plant growth-promoting fungi (PGPF) are a diverse group of nonpathogenic fungi that are associated with plant and influence plant formation and productivity [18,19]. According to the reported literature, PGPF can produce plant growth regulators [20], facilitate nutrient absorption [21], trigger resistance, and produce metabolites for a wide array of deleterious microorganisms [20,22,23,24]. Moreover, soil microbiome contributes to reducing the density of toxic compounds in soils that have harmful effects on plants [25], and enhances the plant’s capability to endure extreme conditions such as salinity, drought and flooding [17].

Tomato (*Solanum lycopersicum* L.), which belongs to the family *Solanaceae*, is one of the most cultivated and consumed vegetables throughout the world [26]. Tomato contains valuable nutrients comprising vitamins, minerals and antioxidants [27], which results in increased consumption and production [28]. However, its production potential has been constrained by a large number of environmental stress factors, and the creation of stress-resistant crops is a main challenge for plant breeding [29]. 

Considering that in nature, plants continuously have to cope with the combination of various abiotic stresses, it is important to provide crops with multiple stress persistence to reduce environmental changes and fulfil the demand for population growth. We hypothesize that *C*. *bertholletiae* could promote tolerance of tomato plants to salinity, drought, and heavy metal stresses. Hence, we investigated whether the application of *C*. *bertholletiae* could boost recovery ability and support the growth of tomatoes under chosen abiotic stresses. Specified aims of this study include: (1) to evaluate the capability of *C*. *bertholletiae* to assist tomato growth under salinity, drought and heavy metal stresses, (2) to examine the effects of *C*. *bertholletiae* applications on numerous physiochemical parameters such as agronomic traits, phytohormone content, sugar content, and antioxidant activity in salinity, drought and heavy metal-stressed plants, (3) to elucidate the modulatory functions of *C*. *bertholletiae* in tomato plants under non-stress and stress conditions, and (4) to authenticate our physiochemical findings via the expression of patterns of different stress-responsive genes comprising *SlACCase* (Acetyl-coenzyme A carboxylase) [30], *SlAOS* (Allene oxide synthase) [31], *SlICS* (Isochorismate synthase*)* [32], *SlCDF3* (Cycling DNA binding with One Finger Factor) [33], *SlGRAS6* (GAI; gibberellic-acid insensitive, RGA; repressor of GAI, SCR; scarecrow) [34], *SlTAF1* (TATA-Box Binding Protein Associated Factor 1) [35], *SlZH13* (Zinc finger-homeodomain protein) [36], *SlRBOHD* (Respiratory burst oxidase homolog protein D) [37], and *SlRING1* (RING E3 ubiquitin ligase) [38]. 

## 2. Results

### 2.1. Identification of Chosen Fungal Strain

**Figure 1:**
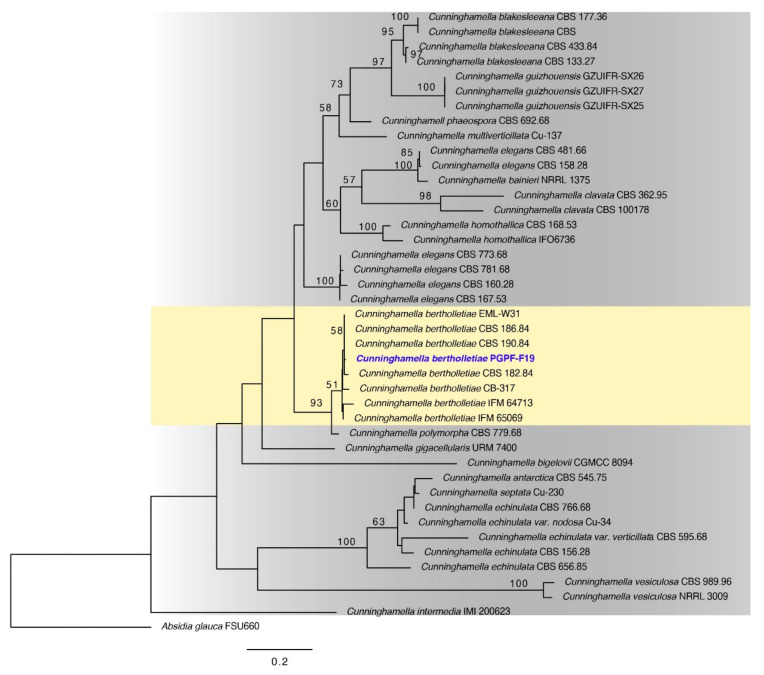
Maximum likelihood tree obtained from the ITS sequence alignment analysis of the species in section *Cunninghamella*. Bootstrap values (>50) are represented by numbers at the nodes based on 1000 replications. The strain in blue font is from our study.

The chosen fungal strain was isolated from the rhizosphere of healthy tomato plants. The phylogenetic analysis, based on combined sequences of ITS (internal transcribed spacer), supported our *Cunninghamella* isolate to be the *Cunninghamella bertholletiae* (Figure 1). 

### 2.2. Indole-3-Acetic Acid, Siderophore and Ammonia Production by C. bertholletiae

We tested some plant growth-promoting properties of the chosen fungal strain, which might take a role in plant growth directly and indirectly (Figure S1). Indole-3-Acetic Acid (IAA) and an ammonia-producing trait were positive for this fungal strain. However, a siderophore-producing trait was negative for the chosen fungal strain. 

### 2.3. Evaluation of C. bertholletiae Stress Resistance on Solid Medium

The fungal strain was inspected in vitro for its capability to endure salt, drought, and heavy metal stress conditions. Ultimate growth tolerance was revealed by the fungal strain under all sodium chloride concentrations (NaCl; 0.5% to 10%), which confirmed it to be supportive for saline-susceptible agricultural regions (Figure S2). Further, the fungal strain was recognized to be resistant towards all the ranges of polyethylene glycol concentrations (PEG 6000 Da; −0.05 to −0.73 MPa). Similarly, the fungal strain demonstrated tolerance to high and low pH (2–12 pH) (Figure S2). Here, we also evaluated the effect of variable ranges of heavy metals on the fungal strain. This strain was found to be resistant to the varied concentrations of cadmium (Cd) and lead (Pb). Ample growth was observed in the presence of 0.4 g/L nickel (Ni) concentration for this fungal strain, whereas at 0.8–1 g/L Ni concentration growth was absolutely eliminated for this strain (Figure S3). Therefore, we can state that this strain can assist in tolerating Ni stress to specific extent. 

### 2.4. Effect of Varied Sodium Chloride Concentrations on Tomato Plant Growth

The impact of various sodium chloride (NaCl) concentrations was examined on tomato plants in order to select the most desirable concentration for subsequent experiments. Applying the slightest NaCl concentration (0.5%) created minimal alteration in relation to the normal plants (Table S1; Figure S4). On the other hand, irrigating tomato plants with 1.5% NaCl resulted in a significant reduction in plant height (35.35%), root length (33.33%), stem diameter (38.18%), leaf length (34.88%), leaf width (38.69%), plant fresh weight (57.29%), plant dry weight (49.20%), root fresh weight (56.41%), root dry weight (44.44%), and leaf number (20.93%) (*p* < 0.05) in comparison to the control plants (Table S1; Figure S4). It should be noted that irrigation with the extreme concentration of NaCl (2.5%) led to a severe reduction in plant growth attributes. Our results demonstrated that 1.5% NaCl caused medium alteration compared to the normal plants and was useful for a meaningful comparison with the plants treated by fungi in further experiments.

### 2.5. Effect of Varied Polyethylene Glycol Concentrations on Tomato Seedling Growth

We recorded numerous plant growth parameters in tomato plants exposed to varying concentrations of polyethylene glycol (PEG 6000 Da; −0.15, −0.3, −0.49 and −0.73 MPa). They typically demonstrated reduction in plant growth characteristics (Table S2; Figure S5). Treatment with the lowest concentration of PEG (−0.15 Mpa) caused minimal modification on growth of tomato plants in comparison with normal plants. However, tomato plants irrigated with the highest PEG concentration (−0.73 Mpa) demonstrated severe and statistically significant reduction in plant growth characteristics. As an example, decreased plant height (41.43%), root length (54.16%), stem diameter (38.18%), leaf length (34.88%), leaf width (36.30%), and chlorophyll content (31.67%) (*p* < 0.05) were noted in PEG-treated plants (−0.73 Mpa) in contrast to control plants (Table S2; Figure S5). These findings show that PEG (−0.73 Mpa) diminished plant growth remarkably. 

### 2.6. Tomato Plants’ Response to PGPF (C. bertholletiae) Inoculant under Varied Abiotic Stresses

#### 2.6.1. Impact of PGPF and Abiotic Stresses on Plant Growth Parameters

**Figure 2:**
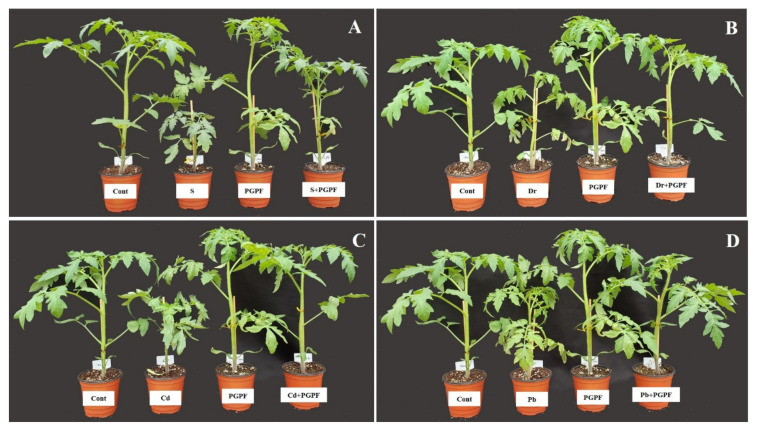
Effects of PGPF inoculation on tomato plant growth under normal and stress conditions after 10 days of treatment (**A**–**D**). Treatments: Cont (control), PGPF (*Cunninghamella bertholletiae*), S (1.5% sodium chloride), S (1.5% sodium chloride) + PGPF (*Cunninghamella bertholletiae*), Dr (25% polyethylene glycol), Dr (25% polyethylene glycol) + PGPF (*Cunninghamella bertholletiae*), Cd (3 mM cadmium), Cd (3 mM cadmium) + PGPF (*Cunninghamella bertholletiae*), Pb (3 mM lead), and Pb (3 mM lead) + PGPF (*Cunninghamella bertholletiae*).

**Figure 3:**
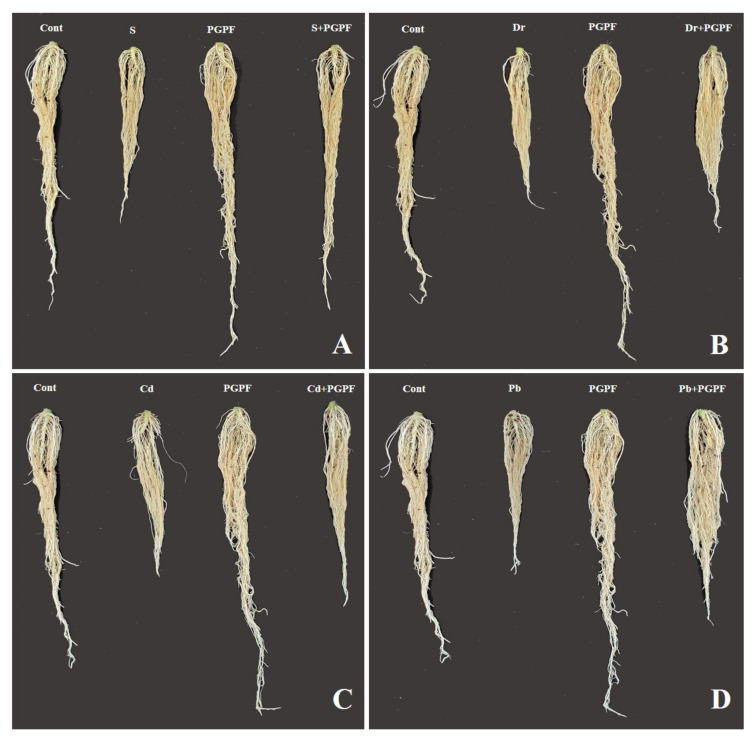
Effects of PGPF inoculation on tomato plant root under normal and stress conditions after 10 days of treatment (**A**–**D**). Treatments: Cont (control), PGPF (*Cunninghamella bertholletiae*), S (1.5% sodium chloride), S (1.5% sodium chloride) + PGPF (*Cunninghamella bertholletiae*), Dr (25% polyethylene glycol), Dr (25% polyethylene glycol) + PGPF (*Cunninghamella bertholletiae*), Cd (3 mM cadmium), Cd (3 mM cadmium) + PGPF (*Cunninghamella bertholletiae*), Pb (3 mM lead), and Pb (3 mM lead) + PGPF (*Cunninghamella bertholletiae*).

The abiotic stresses led to a reduction in several growth parameters as compared to unstressed and untreated tomato plants (Figure 2 and Figure 3, Table 1). On the other hand, some growth parameters were enhanced in PGPF-treated plants. Notably, plant height was improved by 50.98%, 26.41%, 42.59% and 26.41% in the salinity, drought, Cd, and Pb treatments, correspondingly, as opposed to the related heights of untreated stressed plants (*p* < 0.05). In PGPF-treated plants, the plant fresh weight increased by 54.36%, 38.55%, 29.69%, and 51.46% in the salinity, drought, Cd, and Pb treatments, correspondingly, in contrast to the fresh weight of the normal plants (Table 1).

#### 2.6.2. Changes in Chlorophyll and Carotenoid Content

**Figure 4:**
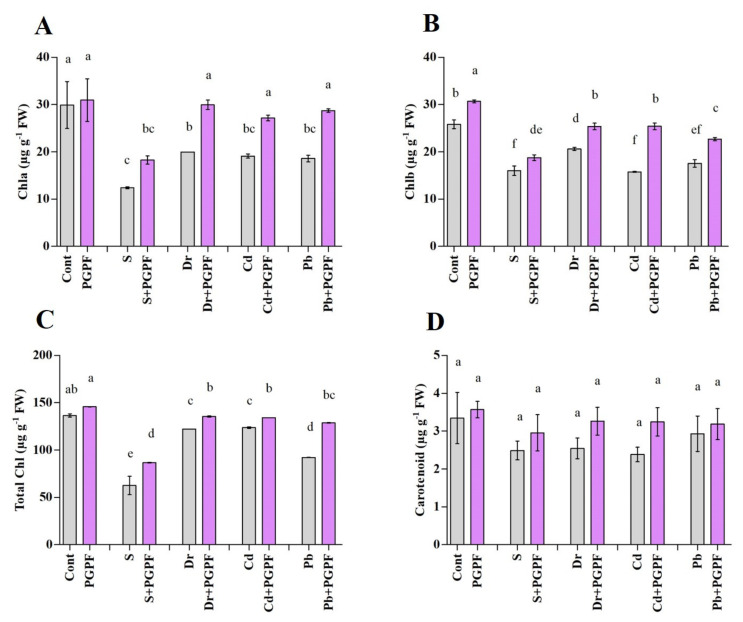
(**A**) chlorophyll a (Chla), (**B**) chlorophyll b (Chlb), (**C**) total chlorophyll (total Chl), and (**D**) carotenoid contents in leaves of tomato plants grown under normal and stress conditions and inoculated with PGPF for 10 days. Treatments: Cont (control), PGPF (*Cunninghamella bertholletiae*), S (1.5% sodium chloride), S (1.5% sodium chloride) + PGPF (*Cunninghamella bertholletiae*), Dr (25% polyethylene glycol), Dr (25% polyethylene glycol) + PGPF (*Cunninghamella bertholletiae*), Cd (3 mM cadmium), Cd (3 mM cadmium) + PGPF (*Cunninghamella bertholletiae*), Pb (3 mM lead), and Pb (3 mM lead) + PGPF (*Cunninghamella bertholletiae*). Values are shown as the means ± SD (*n* = 5) and significant differences at *p* < 0.05 (Tukey test) are indicated by different lowercase letters above the columns.

Chlorophyll a (Chl a) and chlorophyll b (Chl b) contents were enhanced in PGPF-treated stressed plants in comparison to the untreated stressed plants (Figure 4A,B). Similarly, increased contents of total chlorophyll content (total Chl) were recorded in PGPF-treated stressed plants compared to the control stressed plants (Figure 4C). A decline in total Chl was perceived in salinity, drought, Cd, and Pb-stressed plants (54.14%, 10.54%, 9.44%, and 32.45%, correspondingly), which was not the case in the control plants. Conversely, the PGPF application was effective (*p* < 0.05) and helped to enhance total Chl contents under salinity (27.86%), drought (9.73%), Cd (7.81%), and Pb (28.36%) stress conditions, correspondingly (Figure 4C).

#### 2.6.3. Abscisic Acid Response to Abiotic Stresses

**Figure 5:**
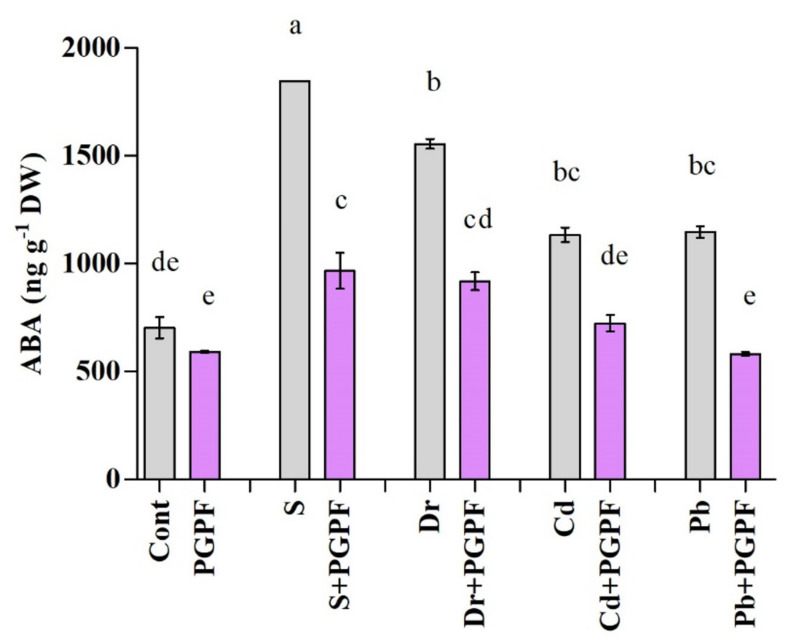
Abscisic acid (ABA) content in leaves of tomato plants grown under normal and stress conditions and inoculated with PGPF for 10 days. Treatments: Cont (control), PGPF (*Cunninghamella bertholletiae*), S (1.5% sodium chloride), S (1.5% sodium chloride) + PGPF (*Cunninghamella bertholletiae*), Dr (25% polyethylene glycol), Dr (25% polyethylene glycol) + PGPF (*Cunninghamella bertholletiae*), Cd (3 mM cadmium), Cd (3 mM cadmium) + PGPF (*Cunninghamella bertholletiae*), Pb (3 mM lead), and Pb (3 mM lead) + PGPF (*Cunninghamella bertholletiae*). Values are shown as the means ± SD (*n* = 5) and significant differences at *p* < 0.05 (Tukey test) are indicated by different lowercase letters above the columns.

Alteration in the content of endogenous abscisic acid (ABA) is exhibited in Figure 5. Salinity, drought and heavy metal stresses considerably enhanced ABA contents in tomato plants. PGPF association with tomato plants diminished ABA level as compared to the level in control plants not exposed to abiotic stresses. Following exposure to salinity, drought, Cd, and Pb stresses, PGPF-treated plants demonstrated a noticeably diminished content of ABA, i.e., 47.62%, 40.94%, 36.18%, and 49.28%, correspondingly (Figure 5).

#### 2.6.4. Alteration in Amino Acid Content

**Figure 6:**
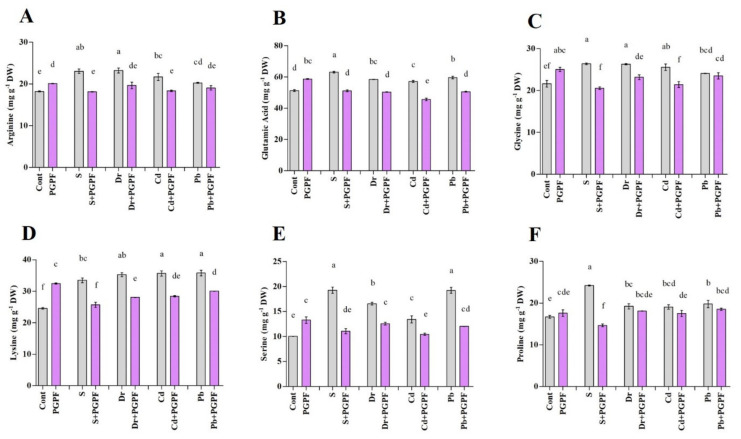
Changes in amino acid contents ((**A**) Arginine, (**B**) Glutamic acid, (**C**) Glycine, (**D**) Lysine, (**E**) Serine, and (**F**) Proline) in leaves of tomato plants grown under normal and stress conditions and inoculated with PGPF for 10 days. Treatments: Cont (control), PGPF (*Cunninghamella bertholletiae*), S (1.5% sodium chloride), S (1.5% sodium chloride) + PGPF (*Cunninghamella bertholletiae*), Dr (25% polyethylene glycol), Dr (25% polyethylene glycol) + PGPF (*Cunninghamella bertholletiae*), Cd (3 mM cadmium), Cd (3 mM cadmium) + PGPF (*Cunninghamella bertholletiae*), Pb (3 mM lead), and Pb (3 mM lead) + PGPF (*Cunninghamella bertholletiae*). Values are shown as the means ± SD (*n* = 5) and significant differences at *p* < 0.05 (Tukey test) are indicated by different lowercase letters above the columns.

The abiotic stresses resulted in accumulations in amino acid content in tomato plants over 10 days (Figure 6A–F). For example, proline content escalated by 31.0%, 13.03%, 12.25%, and 15.51% in salinity, drought, Cd, and Pb-stressed plants, respectively, as compared with the plants under normal conditions. PGPF treatment caused increased amino acid content in unstressed plants in relation to the control plants. In contrast, 10 days after the application of PGPF on damaged plants, amino acid levels (arginine, glutamic acid, glycine, lysine, serine and proline) were demonstrated at the lower level in salinity, drought, Cd, and Pb-stressed plants (Figure 6A–F). For instance, plants treated with PGPF demonstrated a reduction in proline content. Proline levels reduced by 39.43%, 5.81%, 8.07%, and 6.34% in PGPF-treated plants under salinity, drought, Cd, and Pb stress conditions, correspondingly, compared to the stressed plants alone (Figure 6F). The completed outcomes imply that PGPF application reduced amino acid content in tomato plants under stress.

#### 2.6.5. Hydrogen Peroxide and Malondialdehyde Concentrations after Varied Treatments

**Figure 7:**
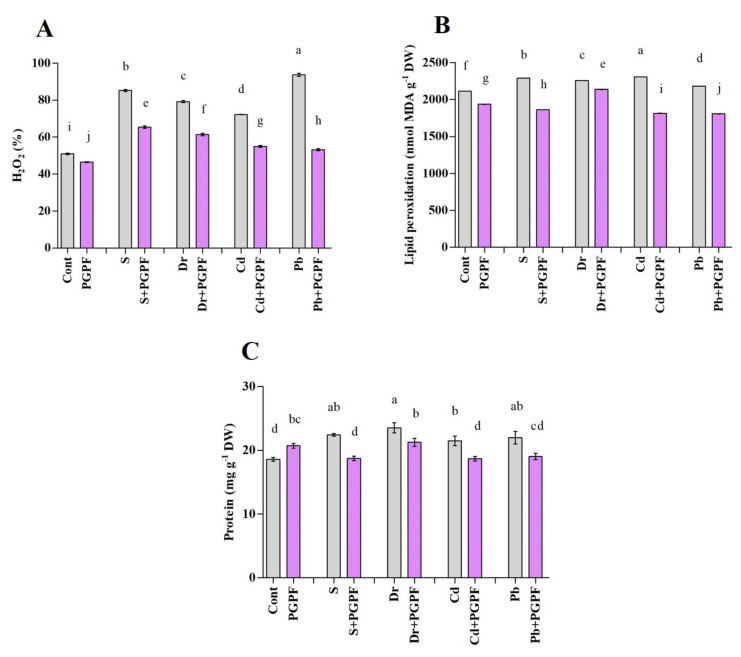
(**A**) Hydrogen peroxide (H_2_O_2_), (**B**) Malondialdehyde (MDA), and (**C**) Protein contents in leaves of tomato plants grown under normal and stress conditions and inoculated with PGPF for 10 days. Treatments: Cont (control), PGPF (*Cunninghamella bertholletiae*), S (1.5% sodium chloride), S (1.5% sodium chloride) + PGPF (*Cunninghamella bertholletiae*), Dr (25% polyethylene glycol), Dr (25% polyethylene glycol) + PGPF (*Cunninghamella bertholletiae*), Cd (3 mM cadmium), Cd (3 mM cadmium) + PGPF (*Cunninghamella bertholletiae*), Pb (3 mM lead), and Pb (3 mM lead) + PGPF (*Cunninghamella bertholletiae*). Values are shown as the means ± SD (*n* = 5) and significant differences at *p* < 0.05 (Tukey test) are indicated by different lowercase letters above the columns.

Abiotic stresses resulted in enhancement in hydrogen peroxide (H_2_O_2_) contents in tomato plants (Figure 7A). H_2_O_2_ amount was increased by 40.21%, 35.68%, 29.51%, and 45.64% under salinity, drought, Cd, and Pb stresses, respectively, as compared with unstressed plants. However, PGPF treatment diminished H_2_O_2_ content in stressed plants; utmost reductions of 23.28%, 22.52%, 23.86%, and 43.23% in H_2_O_2_ contents were recorded in PGPF-treated plants under salinity, drought, Cd, and Pb stresses, correspondingly (*p* < 0.05).

Abiotic stress conditions increased malondialdehyde (MDA) content in untreated stressed tomato plants (Figure 7B); MDA content increased by 7.70%, 6.47%, 8.39%, and 3.15% under salinity, drought, Cd, and Pb stress conditions, respectively. Compared with the untreated stressed plants, the reduced MDA content in PGPF-treated plants was approximately 18.60% under salinity, 5.42% under drought, 21.31% under Cd, and 17.15% under Pb stress conditions (*p* < 0.05).

#### 2.6.6. Changes in Protein and Sugar Content

There was a 10.29% increase of protein content upon PGPF application as compared with untreated control plants. Moreover, the protein content increased by 17.02%, 20.97%, 13.37%, and 15.39% under salinity, drought, Cd, and Pb stresses, correspondingly, compared with the protein content in unstressed plants (*p* < 0.05) (Figure 7C). However, a reduction in protein contents was recorded in PGPF-treated plants during abiotic stress exposure (salt, 16.43%; PEG, 9.65%; Cd, 12.95%; Pb, 13.44%).

**Figure 8:**
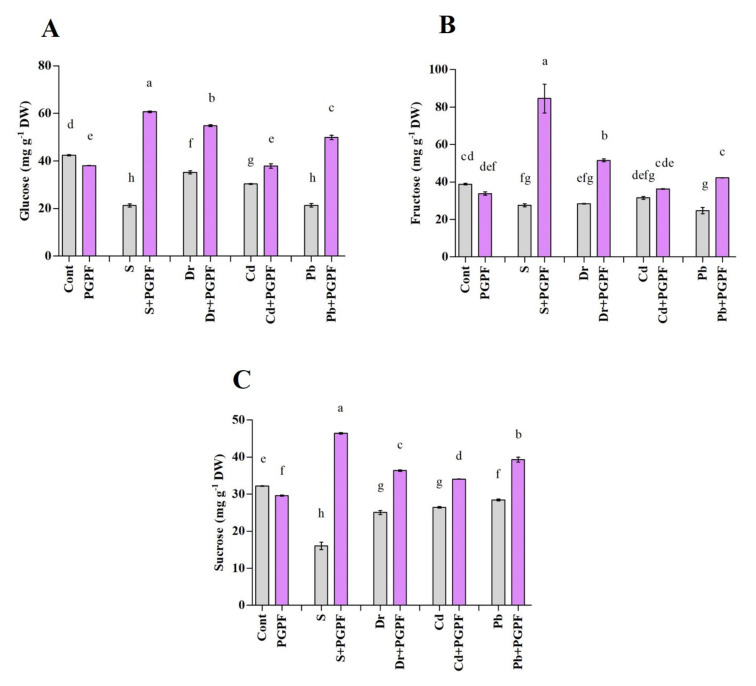
(**A**) Glucose, (**B**) Fructose, and (**C**) Sucrose contents in leaves of tomato plants grown under normal and stress conditions and inoculated with PGPF for 10 days. Treatments: Cont (control), PGPF (*Cunninghamella bertholletiae*), S (1.5% sodium chloride), S (1.5% sodium chloride) + PGPF (*Cunninghamella bertholletiae*), Dr (25% polyethylene glycol), Dr (25% polyethylene glycol) + PGPF (*Cunninghamella bertholletiae*), Cd (3 mM cadmium), Cd (3 mM cadmium) + PGPF (*Cunninghamella bertholletiae*), Pb (3 mM lead), and Pb (3 mM lead) + PGPF (*Cunninghamella bertholletiae*). Values are shown as the means ± SD (*n* = 5) and significant differences at *p* < 0.05 (Tukey test) are indicated by different lowercase letters above the columns.

A considerable decrease in sucrose, glucose and fructose contents was perceived in tomato plants after exposure to stress conditions (Figure 8A–C). For example, sucrose content diminished clearly in response to salinity (50.10%), drought (22.12%), Cd (17.88%), and Pb (11.50%) stresses once compared with the sucrose content of unstressed control plants (Figure 8A–C). PGPF treatment led to decreased sucrose, glucose and fructose contents in unstressed plants in relation to the control plants. In contrast, applying PGPF to stressed plants contributed to a rise in sucrose, glucose and fructose contents. For instance, sucrose content was enhanced by 65.43%, 31.13%, 22.48%, and 27.61% under salinity, drought, Cd, and Pb stresses, correspondingly, compared to the stressed plants alone (Figure 8A–C).

#### 2.6.7. The Activity of Enzymatic and Non-Enzymatic Antioxidants 

**Figure 9:**
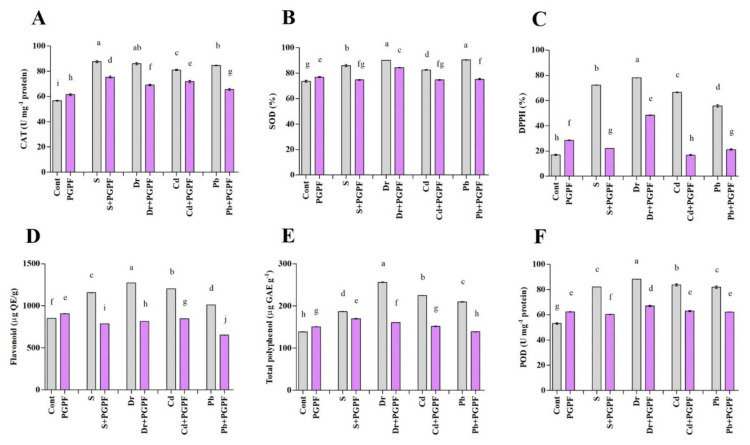
Antioxidant contents ((**A**) CAT (Catalase), (**B**) SOD (Superoxide dismutase), (**C**) DPPH (2,2-diphenyl-1-picrylhydrazyl), (**D**) Flavonoids, (**E**) Total polyphenol and (**F**) POD (Peroxidase)) in leaves of tomato plants grown under normal and stress conditions and inoculated with PGPF for 10 days. Treatments: Cont (control), PGPF (*Cunninghamella bertholletiae*), S (1.5% sodium chloride), S (1.5% sodium chloride) + PGPF (*Cunninghamella bertholletiae*), Dr (25% polyethylene glycol), Dr (25% polyethylene glycol) + PGPF (*Cunninghamella bertholletiae*), Cd (3 mM cadmium), Cd (3 mM cadmium) + PGPF (*Cunninghamella bertholletiae*), Pb (3 mM lead), and Pb (3 mM lead) + PGPF (*Cunninghamella bertholletiae*). Values are shown as the means ± SD (*n* = 5) and significant differences at *p* < 0.05 (Tukey test) are indicated by different lowercase letters above the columns.

Increased superoxide dismutase (SOD) and catalase (CAT) activity was detected in tomato plants in response to stress conditions; whereas, SOD and CAT activity was decreased in PGPF-treated plants affected by the salinity, drought, Cd, and Pb stresses (Figure 9A,B).

DPPH (2,2-diphenyl-1-picrylhydrazyl) activity in tomato plants increased under stress conditions, and DPPH activity reduced in PGPF-treated plants subjected to the same stresses. Precisely, PGPF application resulted in 69.50%, 37.96%, 74.67%, and 61.98% mitigation in the DPPH content under salinity, drought, Cd, and Pb stresses in reference to the DPPH content detected in untreated stressed plants (Figure 9C). 

In addition, flavonoid and total polyphenol function showed a significant increase under stress conditions, but they reduced under these deleterious conditions upon PGPF application (Figure 9D,E). The same trend was noticed with peroxidase (POD), which was enhanced under stress conditions. PGPF treatment mitigated POD content under stress conditions; for example, POD activity reduced by 26.42%, 23.97%, 24.71%, and 24.07% in PGPF-treated plants subjected to salinity, drought, Cd, and Pb stresses (*p* < 0.05), correspondingly. (Figure 9F).

#### 2.6.8. Nutrient, Sodium and Heavy Metal Contents in Plants

Six elements; calcium (Ca), potassium (K), phosphorus (P), sodium (Na), cadmium (Cd) and lead (Pb) were inspected in tomato plants to investigate the impacts of the PGPF inoculant on the nutrient value of tomato plants and its detoxifying function (Table 2). In unstressed plants, a rise was observed in the concentrations of Ca, K and P in plants treated with PGPF compared with the relevant concentrations in control plants. Furthermore, we observed enhancements in K and P contents and a reduction in Ca content in PGPF-treated plants under stress conditions. Increases in Na, Cd and Pb accumulations were recorded in tomato plants under salinity (73.96%) and heavy metal (Cd; 100%, Pb; 100%) stresses in comparison with control plants. However, the PGPF application remarkably diminished the Na accumulation (50.51%) in tomato plants exposed to salinity stress as compared with untreated salinity-stressed plants. Moreover, Cd and Pb accumulations decreased by 67.98% and 54.01%, respectively, in PGPF-treated plants subjected to heavy metal stresses in comparison to stressed plants alone. 

### 2.7. Expression of Salinity-, Drought- and Heavy Metal-Responsive Genes during the C. bertholletiae Application 

Nine genes were assessed for their altered expression in tomato plants under PGPF implementation and abiotic stresses.

#### 2.7.1. Ethylene (*SlACCase*), Jasmonic Acid (*SlAOS*) and Salicylic Acid (*SlICS*) Biosynthesis Genes

**Figure 10:**
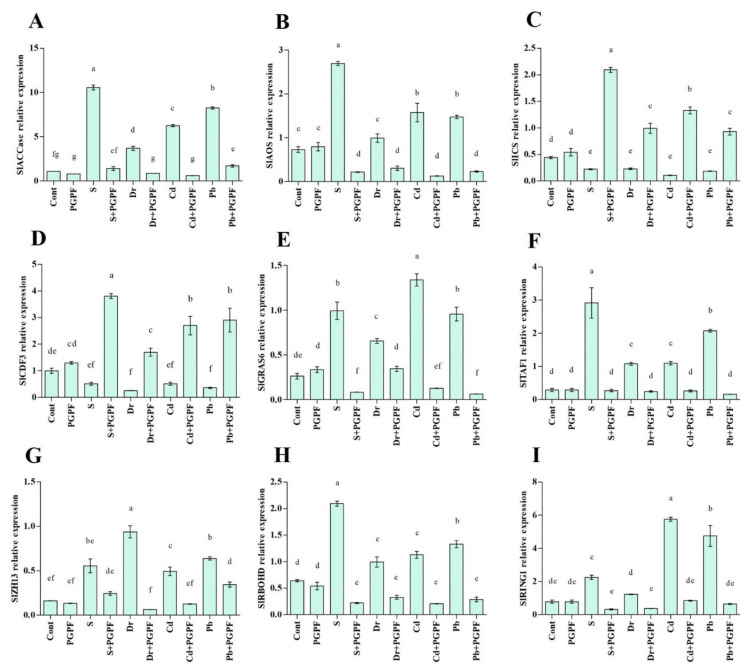
Real-time expression analysis of (**A**) *SlACCase*, (**B**) *SlAOS*, (**C**) *SlICS*, (**D**) *SlCDF3*, (**E**) *SlGRAS6*, (**F**) *SlTAF1*, (**G**) *SlZH13*, (**H**) *SlRBOHD*, and (**I**) *SlRING1* in leaves of tomato plants grown under normal and stress conditions and inoculated with PGPF for 10 days. Treatments: Cont (control), PGPF (*Cunninghamella bertholletiae*), S (1.5% sodium chloride), S (1.5% sodium chloride) + PGPF (*Cunninghamella bertholletiae*), Dr (25% polyethylene glycol), Dr (25% polyethylene glycol) + PGPF (*Cunninghamella bertholletiae*), Cd (3 mM cadmium), Cd (3 mM cadmium) + PGPF (*Cunninghamella bertholletiae*), Pb (3 mM lead), and Pb (3 mM lead) + PGPF (*Cunninghamella bertholletiae*). Values are shown as the means ± SD (*n* = 5) and significant differences at *p* < 0.05 (Tukey test) are indicated by different lowercase letters above the columns.

In the current experiment, we assessed the *SlACCase* (acetyl-CoA carboxylase) transcription pattern in tomato plants. An increase in the *SlACCase* expression level was recorded in salinity, drought, and heavy metal-stressed plants as compared to the expression in unstressed plants. Although the *SlACCase* expression level increased amidst salinity, drought, Cd, and Pb-stressed plants, the PGPF application reduced the *SlACCase* expression by 86.57%, 76.07%, 90.07%, and 79.16%, respectively in stressed plants (Figure 10A). 

Exposure of plants to selected abiotic stresses increased their *SlAOS* gene expression in tomato plants, and PGPF treatment reduced the expression of this gene (Figure 10B). As an example, PGPF application reduced *SlAOS* gene expression by about 91.96%, 69.37%, 91.82%, and 84.48% under salinity, drought, Cd, and Pb stresses, respectively in comparison to stressed plants alone.

The effects of abiotic stresses and PGPF application on *ICS* (isochorismate synthase) was evaluated in tomato plants through change in the *ICS* gene expression (*SlICS*) (Figure 10C). During normal conditions, a few variations were observed in the *SlICS* gene expression in control and PGPF-treated plants; conversely, reduced *SlICS* expression was detected in stressed plants. PGPF-treated plants showed an increase in *SlICS* expression as compared to untreated stressed plants (89.34% under salinity, 76.71% under drought, 91.83% under Cd, and 79.71% under Pb, detrimental statuses).

#### 2.7.2. Transcription Factors (*SlCDF3*, *SlGRAS6*, *SlTAF1*, *SlZH13*)

The findings of the current study demonstrated a reduced *SlCDF3* expression in tomato plants under salinity, drought, Cd, and Pb stresses compared to the control plants. On the other hand, tomato plants treated with PGPF demonstrated enhanced upregulation of *SlCDF3*. A higher *SlCDF3* expression level was detected in PGPF-treated salt (86.48%), drought (84.93%), Cd (80.93%), and Pb (87.75%) stressed plants in comparison with the untreated stressed plants (Figure 10D). 

As shown in Figure 10E, stressed plants demonstrated 73.61% (salinity), 60.02% (drought), 80.41% (Cd), and 72.58% (Pb) enhancements in *SlGRAS6* expression in comparison with the expression detected in control plants. In contrast, reduced expression of *SlGRAS6* was detected in PGPF-treated plants affected by abiotic stresses. PGPF treatment reduced the *SlGRAS6* expression by 91.72% under salinity, 47.61% under drought, 90.61% under Cd, and 93.49% under Pb stresses as compared to the untreated stressed plants (Figure 10E). 

We examined the *SlTAF1* transcription pattern in tomato plants under normal and stress conditions. Enhanced *SlTAF1* expression was detected in tomato plants affected by abiotic stresses. The *SlTAF1* expression level increased by 89.95% (salinity), 72.88*%* (drought), 73.34*%* (Cd), and 85.91% (Pb) in stressed plants (Figure 10F). In addition*,* PGPF-treated tomato plants demonstrated reduced *SlTAF1* expression under stress conditions. Under PGPF application, stressed plants exhibited 90.55% (salinity), 77.05% (drought), 75.92% (Cd), and 92.09% (Pb) lower *SlTAF1* expression compared to the untreated stressed plants. 

The results related to the transcription factor, the *SlZH13* expression level is shown in Figure 10G. A rise in *SlZH13* expression level was detected in salinity, drought, Cd, and Pb-stressed plants by 70.83%, 82.73%, 67.13%, and 74.60%, correspondingly. However, PGPF application decreased the *SlZH13* expression level in salinity (56.18%), drought (93.37%), Cd (74.52%), and Pb (46.17%) compared to the untreated stressed tomato plants.

#### 2.7.3. Reactive Oxygen Species Production (*SlRBOHD*) and E3 Ubiquitin Ligases Activity (*SlRING1*)

This study determined the expression patterns of genes involved in reactive oxygen species production (*SlRBOHD*) and E3 ubiquitin ligases activity (*SlRING1*) in tomato plants. These genes showed varied expression patterns in tomato plants under abiotic stresses and PGPF applications. The *SlRBOHD* expression level was increased in salinity (69.33%), drought (35.33%), Cd (43.15%), and Pb (51.70%) in stressed plants in comparison with normal plants. PGPF-treated stressed plants displayed a mitigation in this gene expression level. The *SlRBOHD* expression level decreased by 89.34% (salinity), 66.64% (drought), 81.53% (Cd), and 78.29% (Pb) in PGPF-treated stressed plants (Figure 10H).

The effect of salinity, drought, Cd, and Pb stresses and PGPF application on RING finger protein (*SlRING1*) was evaluated in tomato plants by examining the expression of this gene (Figure 10I). Expression patterns of this gene in control and PGPF-treated plants demonstrated no variations under unstressed conditions. However, higher *SlRING1* expression was detected in stressed plants. PGPF-treated plants demonstrated a reduction in *SlRING1* expression compared to untreated stressed plants (85.42% under salinity, 69.16% under drought, and 85.17% under Cd, and 86.26% under Pb stress conditions).

## 3. Discussion

We have investigated the effects of *C*. *bertholletiae* on tomato plants. PGPF-inoculated plants appeared to be recovered, and we perceived that PGPF application ameliorates plant growth and development. Additionally, it improves salt, drought, and heavy metal endurance as noticed from the improved growth parameters. Our findings revealed that plants receiving PGPF treatments (unstressed and stressed) maintained greater height, root length, leaf area, plant/root weight, and number of leaves compared to stressed plants in the absence of PGPF. In addition, chlorophyll and carotenoid contents increased in the PGPF-treated plants. Furthermore, we noticed that PGPF application enhanced K and P contents and reduced Ca, Na, Cd, and Pb contents relative to the effects detected in untreated stressed plants. These improvements could be due to higher absorption of nutrients from the rhizosphere, which assists in maintaining plant development and improvement procedure linked to photosynthesis and other metabolisms [39]. 

DOF (DNA binding with One Finger) proteins are plant-specific transcription factors involved in plant growth and responses to abiotic stresses [33]. Indeed, previous studies confirmed its functional roles in germination, seed development, flowering, maturation, plant hormone signaling, and defense responses [40,41,42,43,44]. DOF genes can be categorized into four main clusters of genes called A-D [45]. The D group of DOF genes comprises a group whose transcripts fluctuate under light condition and for this reason they are known as cycling DOF factors (CDFs) [46]. A study undertook by Corrales*,* et al. [33] demonstrated that the expression level of specific DOF genes, *CDF1* and *CDF3* are regulated by various environmental conditions. We explored the expression pattern of *CDF3* in tomato plants subjected to varied abiotic stresses. PGPF-treated plants showed enhanced *CDF3* expression and improved endurance to salinity, drought, Cd, and Pb stresses compared with stressed plants alone. These findings suggest that this gene might participate in abiotic responses which were consistent with a previous report [33]. We confirmed that the improved expression of *CDF3* caused better endurance to chosen abiotic stresses, as demonstrated by survival rates and the other assays. 

Phytohormones are modulators of plant performance, growth modulation and can increase plant adaptation in diverse environmental conditions [47]. The role of abscisic acid in the control of stomatal closure and responses to abiotic stress is strongly confirmed and has been comprehensively studied [48]. Salicylic acid is involved in several plant physiological processes, such as photosynthesis, protection responses, and proline metabolism [49]. It has been shown that salicylic acid improves plant tolerance to the main abiotic stresses comprising salinity [50], drought [51], and heavy metal [52]. The initial pathway for salicylic acid biosynthesis begins from chorismate, which is transformed to isochorismate by isochorismate synthase (ICS) [32]. Yasuda*,* et al. [53] demonstrated that incompatible interaction happens within salicylic acid and abscisic acid pathways. Our biochemical and gene expression outcomes alluded to those abiotic stresses that eliminate salicylic acid levels but boost abscisic acid levels, which is consistent with former reports [54,55,56]. Our outcomes indicate that applying PGPF promotes the capability of tomato plants to withstand a stressful environment by eliminating the abscisic acid contents and augmenting the salicylic acid content.

Plant hormones, ethylene and jasmonic acid have essential roles in several phases of the plant life cycle and they modulate plant responses to abiotic and biotic stresses [57,58]. These unique signaling molecules regulate many developmental and physiological processes in plants, including vegetative growth, seed germination, cell elongation, leaf senescence, stomatal opening, and fruit ripening [58]. Different kinds of external factors such as wounding, micronutrient toxicity, flood, chilling injury, and pollutants enhance the production of ethylene [59,60,61]. Typically, the jasmonic acid signaling pathway cooperatively crosstalks with the ethylene signaling pathway against unfavorable conditions [62]. The results presented here showed enhanced expression of ethylene-responsive gene (*ACCase*) and jasmonic acid biosynthesis gene (*SlAOS*) under salinity, drought and heavy metal stresses which was consistent with previous studies [30,37,63]. PGPF application led to the downregulation of the ethylene-responsive gene and jasmonic acid biosynthesis gene. Research conducted by Habben*,* et al. [64] showed that downregulating of the ethylene biosynthetic pathway can improve the crop yield under abiotic stress conditions. It has been reported that overexpression of AOS leads to the synthesis of jasmonates and therefore enhances drought tolerance [65].

Zinc finger-homeodomain proteins (ZF-HDs) are considered transcription factors that modulate plant developmental processes, and abiotic/biotic stress responses and tolerance [36]. Various studies concentrated on the abiotic stress responses of ZF-HDs have been performed in different plants [66]. In this study, the expression of the ZF-HD gene (*SlZH13*) was strongly induced by salinity, drought and heavy metal stresses and led to its accumulation. Previous studies showed enhanced upregulation of ZF-HD genes under various abiotic stresses such as drought, salinity, cold and ABA treatments [36,66,67]. On the other hand, PGPF application repressed ZF-HD gene expression in tomato plants under stress conditions. This could be due to the stress-relieving impact of PGPF. 

Plants reside in a harsh environment that imposes huge stresses on their growth and productivity. Consequently, they have established sophisticated ways to evade or persist the disastrous consequences. The GRAS protein family (name derived from GAI, RGA, SCR) usually function as a transcription factor and play a significant role under abiotic and biotic stresses [34]. The GRAS family genes have a role in plant growth, gibberellin signal transduction, shoot meristem formation, and radial root patterning [68,69,70,71]. Moreover, they have been connected to abiotic stress responses and plant disease endurance [72,73,74]. We observed enhanced expression of *GRAS* under salinity, drought and heavy metal stresses, whereas PGPF application reduced *GRAS* accumulation in stressed plants. Mayrose*,* et al. [72] showed that *GRAS* accumulation following mechanical and biotic stresses was partly dependent on the signaling molecule jasmonic acid. In addition, it has been reported that *GRAS* was induced in tobacco plants upon hydrogen peroxide treatment [75]. In brief, our findings related to the *GRAS* expression pattern in PGPF-treated plants allow us to presume that PGPF supports stressed plants to cope with countless abiotic stresses.

Detrimental environmental stresses lead to the formation of oxidative stress owing to reactive oxygen species (ROS) generation [76]. At larger concentrations, ROS can induce oxidative damage, modify DNA, cause membrane peroxidation and protein degradation, obstruct metabolic functions, actuate program cell death, and hinder enzymes [77]. Reactive oxygen production is mediated by the *RBOHD* gene, a primary player involved in ROS signaling [37,78]. Considering the current survey, our data revealed induction of *RBOHD* and an obviously higher accumulation of H_2_O_2_ and MDA in stressed plants, which can be attributed to the unbalanced rate of ROS generation and elimination [37,79]. This study suggests that PGPF application reduces the increased H_2_O_2_ and MDA contents in stressed plants at later stages, which is consistent with previous studies [80,81,82,83].

Plants have devised sophisticated procedures to survive and react to stress through alterations at physiological and molecular levels [84]. To combat oxidative damage and sustain the redox balance, plants trigger endogenous procedures that engage enzymatic and non-enzymatic defense mechanisms [85,86]. In this study, the antioxidants’ function increased in stressed plants, but decreased following the use of PGPF in the abiotic stressed plants. Former studies suggested that abiotic stresses could increase or obstruct the expression of antioxidant enzymes [87,88,89,90]. This decline in antioxidant activity suggests that PGPF improves the ability to scavenge excessive ROS, reduces oxidative harm, and enhances endurance to oxidative stress.

Various functions in plants are controlled by protein metabolism and deterioration [91,92]. The protein deterioration pathway of plants comprises two mechanisms, either ubiquitin-mediated proteasome system or autophagy [91]. The ubiquitin proteasome system extensively exists in plant cytoplasm and nucleus and reacts to abiotic stresses by modulating regulatory proteins and deteriorating impaired or misfolded proteins [93]. Under stressful growth conditions, E3 ubiquitin ligases induce the stress signal pathway and improve signal transformation [38,94]. RING finger proteins are recognized for their E3 ligase activity, participate in various physiological processes including cell membrane integrity, ROS regulation, protein function and degradation and regulate the expression of many stress-inducible genes [38,95,96]. In the present study, we observed a higher expression of RING1 under salinity, drought, Cd and Pb stresses which was consistent with previous reports [97]. It is apparent from our outcomes that PGPF application reduces the expression of RING and protein accumulation in stressed plants and improved plant development. 

Sugar molecules have a critical role in elevating protein synthesis, lipid metabolism, and photosynthesis [98]. Adverse environmental conditions can dwindle leaf sugar content and as a consequence provoke physiochemical modifications [99]. It has been confirmed that a high enhancement of sugar in plant demonstrates an immensely protective mechanism against oxidative damage caused by unfavorable conditions [100,101]. In the present study, a noticeable mitigation of glucose, fructose and sucrose was detected in tomato plants inoculated with PGPF under ordinary and unfavorable status. Sugars serve as metabolic resources and they govern countless operations linked with plant performance [98,102]. Findings from this work show that PGPF had a great influence on the sugar accumulation in stressed plants, which possibly performed as an osmoprotectant to govern osmotic adjustments, secure membrane, and scavenge ROS under diverse stress conditions.

Amino acids participate in diverse physiological processes to decrease the damage associated with unfavorable growth conditions [103]. The findings from the current study showed an increased amino acid content in tomato plants under abiotic stresses. This amino acid accumulation may take part in several processes [104]. Previous studies have asserted that amino acid content increases in plants under stress [103,105]. The application of PGPF diminished amino acid contents in stressed plants within the recovery time. An increased level of proline was observed in stressed tomato plants. This could be due to the awakening of proline biosynthesis which ameliorates protein turnover, scavenges reactive oxygen species, and helps the plant to withstand abiotic stress [106,107,108]. Higher proline content has been tightly involved in stabilized membrane and protein structure, minimized cell impairment, and restored plant growth under environmental stress [109,110]. In this study, PGPF application caused reduced amino acid contents, particularly proline content in stressed plants. 

NAC transcription factors are recognized to be involved in plant developmental process and responses against different type of environmental stresses [111]. The expression of TAF1, known as a tomato NAC transcription factor, is triggered in response to abiotic stresses including salinity, drought, extended darkness and abscisic acid, which in turn helps in the adaptation to these stresses [35,112,113]. To evaluate the response of *TAF1* to chosen abiotic stresses, we assessed the impacts of salinity, drought, Cd and Pb on its transcription pattern in tomato plants. These abiotic stresses resulted in a noticeable rise in the *TAF1* expression level. Devkar*,* et al. [114] demonstrated that enhanced expression of *TAF1* during stress controls essential stress regulatory elements comprising stress-responsive transcription factors, ABA biosynthesis gene and signaling, proline accumulation, and the other defense-related elements. PGPF-treated stressed plants exhibited reduced expression of *TAF1*, and subsequently reduced ABA and proline levels, which confirm the stress-mitigating effect of PGPF. 

## 4. Materials and Methods

### 4.1. Collection of Rhizospheric Soil and Isolation of Fungus

Rhizospheric soil was collected from the rhizosphere of healthy tomato plants growing in an agricultural farm stationed at Kyungpook National University (Gunwi-gun), Daegu, South Korea (36°06′48.5″ N 128°38′26.4″ E), following organic cultivation practices. Samples were swiftly placed into Ziplock bags, transported to the laboratory, and isolation was done within 24 h. The fungal strain was isolated from the rhizospheric soil as described by Dey*,* et al. [115]. Rhizospheric soil was collected from root samples by vortexing at high speed (90 s). After that, one gram of collected soil was taken, diluted in sterile distilled water (10 mL) in a test-tube and labelled as stock solution. Finally, the stock solution was subjected to serial dilution and 100 μL of it was added to prepared media and incubated at 27 °C for 7 days. Pure culture was sustained on slant tubes containing potato dextrose agar (PDA) for further analysis.

### 4.2. Molecular Characterizations of the Fungal Strain

Genomic DNA was extracted from fresh fungal culture (seven days old) according to the methods of Al-Sadi*,* et al. [116]. Amplification reactions were carried out using the BioFACT™ 2X Multi-Star PCR Master Mix (BIOFACT, Daejeon, Korea) and a combination of ITS (internal transcribed spacer) primers (ITS1: 5′-TCCGTAGGTGAACCTGCGG-3′/ITS4: 5′-TCCTCCGCTTATTGATATGC-3′) [117] to amplify the ITS region as per the defined conditions [118,119]. The PCR product was examined for the expected size on 1% agarose gel and sequenced at SolGent Co., Ltd. (Daejeon, South Korea). The ITS sequence of the isolate of PGPF was deposited in GenBank (accession number OK175669). The phylogenetic analyses were implemented using the raxml GUI v.1.3 [120] and the tree was created with FigTree v. 1.4.0 (http://tree.bio.ed.ac.uk/software/figtree/, accessed on 11 June 2022).

### 4.3. In Vitro Evaluation of Fungal Strain for Plant Growth-Promoting Traits

Indole-3-Acetic Acid (IAA) production was checked out following the method of Acuña*,* et al. [121]. Luria Bertani broth (LB) incorporated with L-tryptophan (5 mM, LBT), LBT incorporated with 0.05% sodium dodecyl sulfate (0.05%), and glycerol (1%) was used for screening IAA production. After 48 h of growth (25 ± 2 °C), this broth (1.5 mL) was centrifuged (10,000 rpm, 10 min), followed by the addition of 1 mL of Salkawaski reagent (35% perchloric acid and 0.5 M ferric chloride) to the obtained supernatant (1 mL). The emergence of a pink-red color in the test tube validated the IAA production by fungal strain. The siderophore-producing capability was ascertained via chrome azurol S (CAS) agar media as stated by Schwyn and Neilands [122]. The fungal strain was incubated on a CAS agar plate at 25 ± 2 °C for 7 d. The development of an orange-yellow halo around the growth was an indicator of siderophore production. Ammonia production was checked following the method of Szilagyi-Zecchin*,* et al. [123]. Nessler’s reagent was added to the fungal culture, and a development of pale-yellow to dark-orange color confirmed the ammonia production. 

### 4.4. In Vitro Assay of the Fungal Strain for Stress Tolerance

Various abiotic stress tolerance tests (salinity, drought, heavy metals and pH) were performed on different levels with a fresh culture of the isolated fungi. The unaltered medium served as a control. The fungal strain was examined for its intrinsic salt tolerance by checking out its growth on the PDA media, which was amended with diverse concentrations of sodium chloride (NaCl, Sigma-Aldrich, St. Louis, MI, USA) (0.5%, 2.5%, 5%, 7.5%, and 10%). The plates were incubated for 7 d at 25 ± 2 °C. The drought resistance test was assessed by growing the fungal strain on prepared PDA medium supplemented with various osmotic potentials (−0.05, −0.15, −0.3, −0.49, and −0.73 MPa) of polyethylene glycol (PEG-6000 Da, Merck-Schuchardt, Hohenbrunn, Germany) [124]. The heavy metal tolerance of fungal strain was assayed on a PDA medium amended with different cadmium (Cd), lead (Pb), and nickel (Ni) concentrations ranging from 0.4 to 1.0 g L^−1^ at 25 ± 2 °C for 7 d. The considerable growth of fungal strain in the presence of heavy metals amid 7 d at 25 ± 2 °C was taken into account as heavy metal resistance [125]. pH tolerance was examined by incubating the fungal strain at various pH regimes (namely, 2, 4, 6, 8, and 12). 

### 4.5. Greenhouse Experiments

The abiotic stress tolerance strain (*C*. *bertholletiae*) was cultured on PDA for 7 d at 25 ± 2 °C. The inoculum suspension was prepared as stated by Zhang*,* et al. [126]. The spore density was examined with a haemocytometer (Bright-Line^TM^, Sigma-Aldrich, St. Louis, MI, USA), and the final suspension (1.0 × 10^8^ spore/mL) was maintained at 4 °C. 

Tomato seeds (rendered by Danong Co., Ltd., Namyangju, Korea) of the same size and color were immersed in 70% ethanol and 2.5% sodium hypochlorite and rinsed several times in sterile distilled water. Subsequently, the sterile seeds were tested for the sterilization procedure and vitality [127,128]. Seeds (one seed/pot) were planted in trays loaded with horticultural soil (Shinsung Mineral Co., Ltd., Daegu, Chungcheongbuk-do, Korea). After that, trays were kept in a greenhouse and seedlings were grown under natural daylight, with 70% relative humidity and 24 °C/16 °C (day/night) temperature, and irrigated daily. Afterwards, similar seedlings were selected after three weeks, transferred into pots, and received diverse treatments. Whole tomato seedlings were randomly split into three groups in this way: (i) control, irrigated with distilled water, (ii) salinity, irrigated with sodium chloride (0.5, 1, 1.5 and 2.5%), and (iii) drought, irrigated with polyethelene glycol (5%; −0.15 MPa), (10%; −0.3 MPa), (15%; −0.49 MPa), and (25%; −0.73 MPa). Individual treatment consisted of five repetitions. Plants exposed to individual treatment were assessed for different morphological traits amid 10 days. Ultimately, 1.5% sodium chloride and 25% polyethelene glycol (−0.73 MPa) were chosen as the most appropriate concentrations in subsequent experiments. Similarly, we decided to expose tomato plants to 3 mM cadmium (Cd) and lead (Pb) stresses.

### 4.6. Influence of C. bertholletiae on Stressed-Tomato Plants

The tomato seedlings were cultivated in autoclaved-sterilized soil in a greenhouse under the previously mentioned conditions. The obtained seedlings (three weeks’ old) were split into two groups and treated with selected treatment for 10 days: (i) control, treated with sterile distilled water; and (ii) PGPF, treated with fungal suspension. Consequently, the groups of PGPF treated and untreated seedlings were then split into varied groups which are clarified in Table 3. The tomato seedlings were exposed to the chosen treatments for 10 days. In each case, tomato leaves (third or fourth leaf from growing tip) were collected and either promptly used or rapidly deactivated in liquid nitrogen and maintained at −80 °C for further analysis. Soil samples from each treatment were tested at the end of selected period to evaluate soil moisture, pH, and electrical conductivity (EC). (Table S3).

### 4.7. Measurements of Plant Physiological Traits and Chlorophyll Content

Growth assessment was carried out on the fresh tomato plants by estimating several agronomic traits to investigate the influence of one-by-one treatment on the tomato plants. In a preliminary salt and drought screening experiment, chlorophyll concentration in leaves was determined with a SPAD (soil plant analysis development) meter (SPAD-502, Konica Minolta, Tokyo, Japan). The plant and root dry weights were examined by oven-drying them at 60 °C for 48 h [129]. 

### 4.8. Determination of Photosynthetic Pigments and Abscisic Acid 

Photosynthetic pigments, Chlorophyll a (Chl a), chlorophyll b (Chl b) and carotenoid were estimated as described previously [130,131]. Fresh leaf samples (about 100 mg) were placed in a vial filled with dimethyl sulfoxide (5 mL DMSO) and incubated at 65 °C for 3 h. The absorbance of the extraction was recorded using a spectrophotometer (Multiskan^TM^ GO Microplate Spectrophotometer, Thermo Fisher Scientific, Waltham, MA, USA) at a wavelength of 663 nm, 645 nm, and 470 nm for chlorophyll a, b, and carotenoids respectively. 

The endogenous abscisic acid (ABA) content of freeze-dried powder samples (approximately 0.1 g) was extracted based on the defined procedures [132,133]. ABA was extracted from the samples using an extraction solution comprising 95% isopropanol, 5% glacial acetic acid, and ABA standard (20 ng). The filtered extract was concentrated using a rotary evaporator, then dissolved in sodium hydroxide (5 mL, 1 N NaOH), and washed three times with dichloromethane (10 mL CH_2_Cl_2_) to remove lipophilic materials. After adjusting the pH of the aqueous phase to 3.5 by adding hydrochloric acid (6N HCl), ethyl acetate was added to it by vortexing to partition it. The supernatant, ethyl acetate extract, was evaporated to dryness and then dissolved in phosphate buffer (pH 8.0) to remove phenolic compounds. PVPP (polyvinylpolypyrrolidone) was added to the extracted solution (phosphate buffer) and kept on a shaker for 40 min at 150 rpm. The pH of the phosphate buffer was brought to 2.5 and partitioned into ethyl acetate. The ethyl acetate extract was evaporated to dryness. The dried residue was dissolved in dichloromethane (CH_2_Cl_2_), followed by passing through a silica cartridge (Sep-Pak; Water Associates, Milford, MA, USA) which was pre-washed with dichloromethane and diethyl ether methanol (C_5_H_14_O_2_). Eventually, the obtained extract was desiccated and methylated using nitrogen gas and diazomethane (CH_2_N_2_), respectively. Gas chromatography-mass spectrometry (Agilent 6890N Gas Chromatograph, Santa Clara, CA, USA) was exerted to evaluate the ABA amount. The responses to ions (*m*/*z* of 190 and 162 for Me-ABA, and 166 and 194 for Me-[^2^H_6_]-ABA) were detected using Lab-Base (ThermoQuest software, Manchester, UK).

### 4.9. Essential Amino Acid Analysis

This experiment was carried out by hydrolyzing approximately 50 milligrams of freeze-dried powder samples in 1 mL hydrochloric acid (6-N HCl, 24 h, 110 °C) [134]. After that, samples were cooled down to 4 °C and hydrochloric acid was evaporated under a nitrogen stream. The dried samples were dissolved in distilled water (1 mL) and filtered (0.45 µm filter membrane) before being loaded into an Amino Acid analyzer. Amino acid standards were acquired from Sigma-Aldrich (St. Louis, MI, USA) and diluted to desired concentrations. The amino acid composition was examined using Amino Acid analyzer (L-8900 Hitachi High-Technologies Co., Tokyo, Japan). 

### 4.10. Soluble Protein and Sugar Extraction

The estimation of protein content in leaf samples was carried out according to standard procedures [135,136]. The freeze-dried samples (0.1 g) were ground and homogenized in 1 mL phosphate buffer (50 mM, pH 7.0), and subsequently centrifuged (10,000 rpm, 10 min, 4 °C). The collected supernatant was treated with a proper reagent and served as a source for determining protein. The absorbance of the obtained mixture was taken at 595 nm by a spectrophotometer (Multiskan^TM^ GO Microplate Spectrophotometer, Thermo Fisher Scientific, Waltham, MA, USA) with bovine serum albumin as control.

The freeze-dried leaves were ground into powder, and soluble sugar was extracted following the protocol of Zhu*,* et al. [137]. The powdered sample (100 mg) was sonicated in double-distilled water (800 µL, 30 min) and then kept at 80 °C for 30 min with occasional shaking. The mixture was centrifuged (10,000 rpm, 10 min, 4 °C) and the obtained supernatant was vacuum-dried at 60 °C, then dissolved in 80% ethanol. After this, the suspension was centrifuged (10,000 rpm, 10 min, 4 °C) and the obtained supernatant was dried again. The dehydrated remnant was dissolved in acetonitrile and preserved at −20 °C. The high-performance liquid chromatography (HPLC, Waters Co., Milford, MA, USA) Equipped with Alltech 3300 ESLD detector (Alltech, Deerfield, IL, USA) was used to determine soluble sugars in collected residues. Separation was accomplished on an XBridge^TM^ Amide column [4.6 mm × 250 mm, 3.5 µm particle size] (Waters Co., Milford, MA, USA). Prepared samples and standards were passed through a 0.45 µm filter membrane and loaded (20 µL) onto the HPLC machine with fixed conditions (solvent ratio; 85 acetonitrile: 15 water (*v*/*v*), flow rate 0.5 mL min^−1^, column temperature 90 °C). Peak quantification was carried out using the calibration standards of HPLC grade sugars.

### 4.11. Antioxidant Activity in Inoculated and Non-Inoculated Tomato Plants

Polyphenol oxidase (PPO) and peroxidase (POD) activities were assayed in freeze-dried leaves using catechol (50 mM) and pyrogallol (50 mM), respectively [138,139]. Afterwards, their activities were expressed as a change of absorbance at 490 and 430 nm, respectively. The activity of catalase (CAT) was determined according to the described method [140]. The reaction mixture contained phosphate buffer (100 mM, pH 7.0), ethylenediaminetetraacetic acid (EDTA, 0.1 µM), H_2_O_2_ (20 mM) and enzyme extract (50 μL). The mixture absorbance was estimated spectrophotometrically at 240 nm (Multiskan^TM^ GO Microplate Spectrophotometer, Thermo Fisher Scientific, Waltham, MA, USA). Superoxide dismutase (SOD) activity was assayed examining its capacity to inhibit the photochemical reduction of nitro blue tetrazolium chloride (NBT) as described by Chen*,* et al. [141]. The reaction mixture comprised phosphate buffer (50 mM, pH 7.8), EDTA (0.1 µM), methionine (13 mM), NBT (75 μM), riboflavin (2 μM) and enzyme extract (50 μL). The reaction mixture was kept in darkness for 15 min and absorbance was recorded by spectrophotometer at 540 nm. Flavonoid content was determined using aluminum chloride colorimetric method [142] and a spectrometer was used to read the absorbance at 415 nm. DPPH (1,1-diphenyl-2-picrylhydrazyl) activity was determined using the method of Wang*,* et al. [143]. Briefly, freeze-dried leaf samples (0.4 g) were homogenized in absolute ethanol (4 mL). After centrifugation, the supernatant (0.2 mL) was mixed with absolute ethanol (0.8 mL), DPPH (0.5 mM, 1 mL) and acetate buffer (100 mM, 2 mL). Eventually, the absorbance was measured at 517 nm. Total polyphenols were examined with the Folin-Ciocalteu reagent based on a previous report of Zheng and Wang [144] and using gallic acid as a standard. The mixture was incubated at 30 °C for 1.5 h and then the absorbance was measured at 765 nm using a spectrophotometer (Multiskan^TM^ GO Microplate Spectrophotometer, Thermo Fisher Scientific, Waltham, MA, USA).

### 4.12. Hydrogen Peroxide and Lipid Peroxidation Quantification of Tomato Plants 

The previously described method was followed to measure hydrogen peroxide content (H_2_O_2_) in plant extracts [145]. The freeze-dried samples (0.3 g each) were blended with 5 mL 0.1% trichloroacetic acid (TCA) in an ice bath and centrifuged at 12,000 rpm for 20 min. The obtained supernatant (0.5 mL) was combined with 10 mM potassium phosphate buffer (0.5 mL, pH 7.0) and 1 M potassium iodide (1 mL). The reaction mixture intensity was recorded at 390 nm using a spectrophotometer (Multiskan^TM^ GO Microplate Spectrophotometer, Thermo Fisher Scientific, Waltham, MA, USA).

The lipid peroxidation level of freeze-dried samples (0.2 g) was determined by applying the thiobarbituric acid (TBA) test [146]. The mixture contained 0.5 mL of 0.1% TCA extract that was added to 1 mL of 0.5% TBA (prepared in 20% TCA). The mixture was incubated in boiling water (95 °C, 30 min), followed by cooling in an ice bath (10 min). Subsequently, the homogenate was centrifuged (12,000 rpm, 5 min) and the supernatant absorbance was measured at 532 and 600 nm. The lipid peroxidation level was calculated according to a standard curve (Multiskan^TM^ GO Microplate Spectrophotometer, Thermo Fisher Scientific, Waltham, MA, USA).

### 4.13. Assessment of Nutrient, Sodium, Cadmium and Lead Contents in Tomato Plants

Plants grown under varied treatments were selected to determine their nutrient contents. Samples were prepared based on the described method of Choi*,* et al. [147]. Concisely, freeze-dried leaves (50 mg) were powdered and digested in nitric acid (0.6 mL) in a glass test tube at 120 °C for 2 h. Additional digestion of samples were carried out in 60% perchloric acid (0.4 mL, 150–180 °C, 2 h). The resulting samples were cooled to room temperature, diluted in nanopure water (5 mL) and used to evaluate calcium (Ca), potassium (K), phosphorus (P), sodium (Na), cadmium (Cd), and lead (Pb) concentrations in tomato plants employing an inductively-coupled plasma mass spectrometer (Optima 7900DV, Perkin-Elmer, Akron, OH, USA). 

### 4.14. RNA Isolation and Gene Expression Analysis

Total RNA of fresh tomato leaves (100 mg) from different treatments was extracted using the Trizol in conformity with the formerly described method [148]. NanoDrop 2000 spectrophotometer (Thermo Fisher Scientific, Wilmington, DE, United States) was used to examine the RNA quality and concentration. The RNA was reverse-transcribed to cDNA and used for real-time PCR (qRT-PCR) assay. For each sample, cDNA was generated by utilizing a BioFACT RT-Kit (BIOFACT, Daejeon, Korea), and maintained at −20 °C before further analysis by qRT-PCR (Illumina, San Diego, CA, USA). Specific primers used in qRT-PCR analysis are presented in Table S4. 

### 4.15. Data Analysis

SAS statistical software (version 9.4, SAS institute, Cary, NC, USA) was employed to examine the data via ANOVA. Tukey’s test at *p* < 0.05 was used to specify substantial contrasts among treatments. Graphs were portrayed with Origin Pro (version 9.85, Northampton, MA, USA). The data exhibited are the means of five replicates for treatments and control.

## 5. Conclusions

The application of *C*. *bertholletiae* not only enhanced tomato growth under salinity, drought, and heavy metal toxicity, but also unquestionably prompted tomato-plant tolerance to these abiotic stresses. Likewise, *C*. *bertholletiae* improved host biochemistry to eliminate the disastrous consequences of abiotic stresses. In this report, salt, drought, and heavy metal stresses restrained some genes. However, *C*. *bertholletiae* was apt for confronting the repression consequence of salt, drought, and heavy metal stresses through reviving the expression of several repressed genes. *C*. *bertholletiae* affected the expression of stress-related genes, explicitly *SlCDF3*, *SlICS*, *SlACCase*, *SlAOS*, *SlGRAS6*, *SlRBOHD*, *SlRING1*, *SlTAF1*, and *SlZH13*. Generally, the gained outcomes granted evident validation to substantiate the stress-relieving influence of *C*. *bertholletiae*. These outcomes exhibit a peculiar approach for enhancing the performance of tomatoes and possibly other vegetables of economic importance. 

## Figures and Tables

**Table 1:** Effects of PGPF inoculation on tomato plant growth parameters under normal and stress conditions after 10 days of treatment.

Treatment	Plant Height	Root Length	Stem Diameter	Leaf Length	Leaf Width	Plant Fresh Weight	Plant Dry Weight	Root Fresh Weight	Root Dry Weight	No. Leaf
	(cm)	(cm)	(cm)	(cm)	(cm)	(g)	(g)	(g)	(g)	
Cont	27.5 ± 0.75 b	22.75 ± 0.37 b	0.75 ± 0.07 b	24.5 ± 0.25 b	18.5 ± 0.0 ab	24.91 ± 0.45 b	1.22 ± 0.01 b	4.73 ± 0.36 b	0.17 ± 0.01 b	11.0 ± 0.5 ab
PGPF	36.5 ± 0.25 a	27.75 ± 0.87 a	0.87 ± 0.03 a	28.5 ± 0.0 a	19.75 ± 0.87 a	31.57 ± 0.78 a	1.98 ± 0.01 a	5.72 ± 0.86 a	0.28 ± 0.0 a	12.0 ± 0.5 a
S	12.5 ± 0.25 f	14.5 ± 0.25 e	0.34 ± 0.0 f	11.5 ± 0.75 fg	7.25 ± 0.62 f	5.75 ± 0.87 h	0.45 ± 0.02 f	1.62 ± 0.0 c	0.07 ± 0.0 c	6.5 ± 0.25 de
S + PGPF	25.5 ± 0.75 c	22.5 ± 0.22 b	0.48 ± 0.0 e	17.5 ± 1.0 d	14.5 ± 0.25 d	12.6 ± 0.0 ef	1.07 ± 0.03 c	4.25 ± 0.12 b	0.16 ± 0.01 b	10.5 ± 0.0 b
Dr	19.5 ± 0.75 d	13.5 ± 0.65 e	0.32 ± 0.04 f	13.5 ± 1.0 ef	10.5 ± 0.0 e	9.58 ± 0.79 g	0.51 ± 0.04 ef	1.81 ± 0.09 c	0.04 ± 0.0 c	7.5 ± 0.75 cd
Dr + PGPF	26.5 ± 0.25 bc	16.5 ± 0.0 d	0.60 ± 0.0 d	18.75 ± 0.37 d	14.75 ± 0.37 d	15.59 ± 0.79 d	0.91 ± 0.04 d	3.98 ± 0.01 b	0.15 ± 0.0 b	10.5 ± 0.0 b
Cd	15.5 ± 0.75 e	13.5 ± 0.65 e	0.49 ± 0.01 e	11.0 ± 0.5 g	7.5 ± 0.75 f	14.44 ± 0.22 de	0.60 ± 0.0 e	2.42 ± 0.21 c	0.07 ± 0.0 c	6.0 ± 0.5 e
Cd + PGPF	27.0 ± 0.5 bc	17.75 ± 0.87 cd	0.65 ± 0.0 cd	22.5 ± 0.0 bc	17.25 ± 0.62 bc	20.54 ± 0.3 c	1.10 ± 0.05 c	3.98 ± 0.01 b	0.15 ± 0.0 b	10.0 ± 0.5 b
Pb	19.5 ± 0.75 d	13.5 ± 0.65 e	0.32 ± 0.02 f	14.0 ± 0.5 e	10.5 ± 0.25 e	11.91 ± 0.95 f	0.56 ± 0.0 e	2.05 ± 0.02 c	0.08 ± 0.01 c	8.5 ± 0.0 c
Pb + PGPF	26.5 ± 0.5 bc	18.75 ± 0.37 c	0.73 ± 0.0 bc	21.75 ± 0.87 c	16.5 ± 0.0 c	24.54 ± 0.04 b	1.15 ± 0.02 bc	4.3 ± 0.15 b	0.16 ± 0.04 b	11.0 ± 0.5 ab

Treatments: Cont (control), PGPF (*Cunninghamella bertholletiae*), S (1.5% sodium chloride), S (1.5% sodium chloride) + PGPF (*Cunninghamella bertholletiae*), Dr (25% polyethylene glycol), Dr (25% polyethylene glycol) + PGPF (*Cunninghamella bertholletiae*), Cd (3 mM cadmium), Cd (3 mM cadmium) + PGPF (*Cunninghamella bertholletiae*), Pb (3 mM lead), and Pb (3 mM lead) + PGPF (*Cunninghamella bertholletiae*). Values are shown as the means ± SD (*n* = 5) and significant differences at *p* < 0.05 (Tukey test). Data within the same column followed by different lowercase letters are significantly different.

**Table 2:** Calcium (Ca), potassium (K), phosphorus (P), sodium (Na), cadmium (Cd) and lead (Pb) contents in tomato plants grown under stress and control conditions with or without PGPF after 10 days of treatment.

Sample Name	Ca (µg/kg)	K (µg/kg)	P (µg/kg)	Na (µg/kg)	Cd (µg/kg)	Pb (µg/kg)
Cont	8.25 ± 0.12 de	51.18 ± 0.59 h	6.31 ± 0.15 c	4.02 ± 0.01 c	N.D	N.D
PGPF	10.28 ± 0.14 c	59.94 ± 0.97 g	7.05 ± 0.52 bc	4.60 ± 0.3 c	N.D	N.D
S	14.38 ± 0.19 a	64.81 ± 0.4 f	7.51 ± 0.75 abc	15.44 ± 0.72 a	N.D	N.D
S + PGPF	7.22 ± 0.61 e	73.05 ± 0.52 c	8.81 ± 0.4 a	7.64 ± 0.82 b	N.D	N.D
Dr	8.44 ± 0.22 de	60.18 ± 0.09 g	7.79 ± 0.89 abc	4.20 ± 0.1 c	N.D	N.D
Dr + PGPF	5.58 ± 0.79 f	65.84 ± 0.92 ef	8.20 ± 0.1 ab	4.51 ± 0.25 c	N.D	N.D
Cd	13.02 ± 0.51 ab	67.41 ± 0.7 e	6.80 ± 0.4 bc	4.45 ± 0.22 c	2.78 ± 0.05 a	N.D
Cd + PGPF	9.21 ± 0.6 cd	77.93 ± 0.96 a	7.93 ± 0.96 abc	5.0 ± 0.5 c	0.89 ± 0.01 b	N.D
Pb	12.41 ± 0.2 b	70.13 ± 0.06 d	6.83 ± 0.41 bc	4.36 ± 0.18 c	N.D	2.24 ± 0.03 a
Pb + PGPF	8.69 ± 0.34 d	75.33 ± 0.66 b	8.03 ± 0.01 abc	5.27 ± 0.63 c	N.D	1.03 ± 0.01 b

Treatments: Cont (control), PGPF (*Cunninghamella bertholletiae*), S (1.5% sodium chloride), S (1.5% sodium chloride) + PGPF (*Cunninghamella bertholletiae*), Dr (25% polyethylene glycol), Dr (25% polyethylene glycol) + PGPF (*Cunninghamella bertholletiae*), Cd (3 mM cadmium), Cd (3 mM cadmium) + PGPF (*Cunninghamella bertholletiae*), Pb (3 mM lead), and Pb (3 mM lead) + PGPF (*Cunninghamella bertholletiae*), N.D (not detected). Values are shown as the means ± SD (*n* = 5) and significant differences at *p* < 0.05 (Tukey test). Data within the same column followed by different lowercase letters are significantly different.

**Table 3:** Experimental work plan.

Symbol	Treatment
Cont	irrigated with sterile distilled water
PGPF	irrigated with *Cunninghamella bertholletiae*
S	irrigated with sodium chloride (1.5% NaCl)
S + PGPF	irrigated with sodium chloride (1.5% NaCl) + *Cunninghamella bertholletiae*
Dr	irrigated with polyethylene glycol (25%PEG; −0.73 Mpa)
Dr + PGPF	irrigated with polyethylene glycol (25%PEG; −0.73 Mpa) + *Cunninghamella bertholletiae*
Cd	irrigated with cadmium (3 mM Cd)
Cd + PGPF	irrigated with cadmium (3 mM Cd) + *Cunninghamella bertholletiae*
Pb	irrigated with lead (3 mM Pb)
Pb + PGPF	irrigated with lead (3 mM Pb) + *Cunninghamella bertholletiae*

## Data Availability

The data presented in this study are available in the tables and Supplementary Tables.

## Supplementary Materials

The following supporting information can be downloaded at: https://www.mdpi.com/article/10.3390/ijms23168909/s1.
