# Supplementary material for: Effects of the Rhizosphere Fungus Cunninghamella bertholletiae on the Solanum lycopersicum Response to Diverse Abiotic Stresses
Source: Int J Mol Sci. 2022 Aug 10;23(16):8909. doi: 10.3390/ijms23168909 (PMC9408995; doi:10.3390/ijms23168909)
Supplement: Supplementary file 1 [file ijms-23-08909-s001.zip › ijms-1831268-supplementary.pdf]

# Supplementary materials

# Effects of the Rhizosphere Fungus *Cunninghamella bertholletiae* on the *Solanum lycopersicum* Response to Diverse Abiotic Stresses

**Elham Ahmed Kazerooni [1,*], Sajeewa S. N. Maharachchikumbura [2], Abdullah Mohammed Al-Sadi [3], Umer Rashid [4], Il-Doo Kim [1], Sang-Mo Kang [1] and In-Jung Lee [1,*]**

[1] Department of Applied Biosciences, Kyungpook National University, Daegu 41566, Korea
[2] School of Life Science and Technology, Center for Informational Biology, University of Electronic Science and Technology of China, Chengdu 611731, China
[3] Department of Plant Sciences, College of Agricultural and Marine Sciences, Sultan Qaboos University, P.O. Box 34, Al-Khoudh 123, Oman
[4] Institute of Nanoscience and Nanotechnology (ION2), Universiti Putra Malaysia, Serdang 43400, Selangor, Malaysia
* Correspondence: elham.ghasemi.k@gmail.com (E.A.K.); ijlee@knu.ac.kr (I.-J.L.)

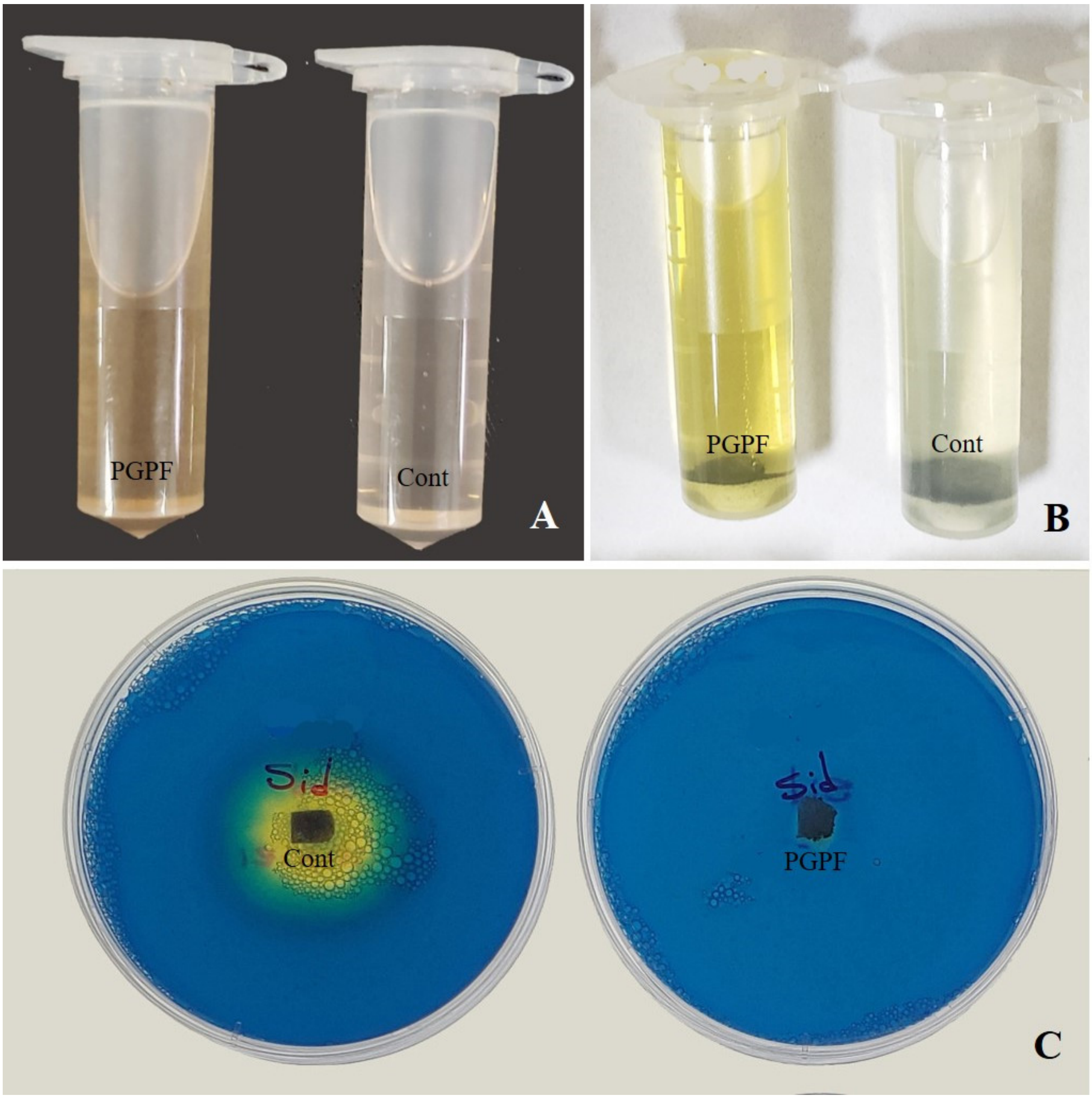


**Figure S1.** (**A**) IAA production, (**B**) Ammonia production, (**C**) Siderophore production of the selected fungal strain (*Cunninghamella bertholletiae*) associated with *Solanum lycopersicum* in this study.

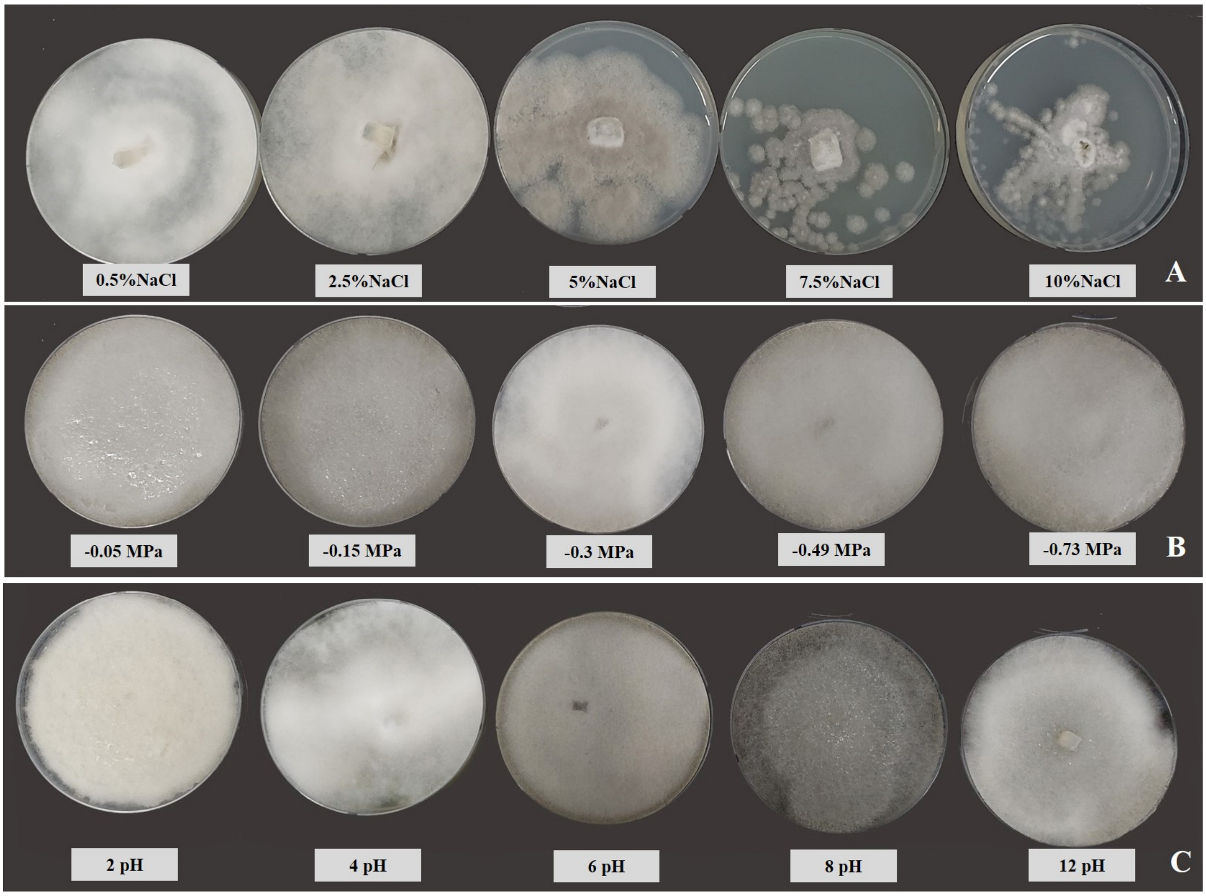


**Figure S2.** (**A**) Salinity (sodium chloride; NaCl), (**B**) Drought (polyethelene glycol; PEG 6000 Da) and (**C**) pH tolerance ability of the selected fungal strain (*Cunninghamella bertholletiae*) associated with *Solanum lycopersicum* in this study.

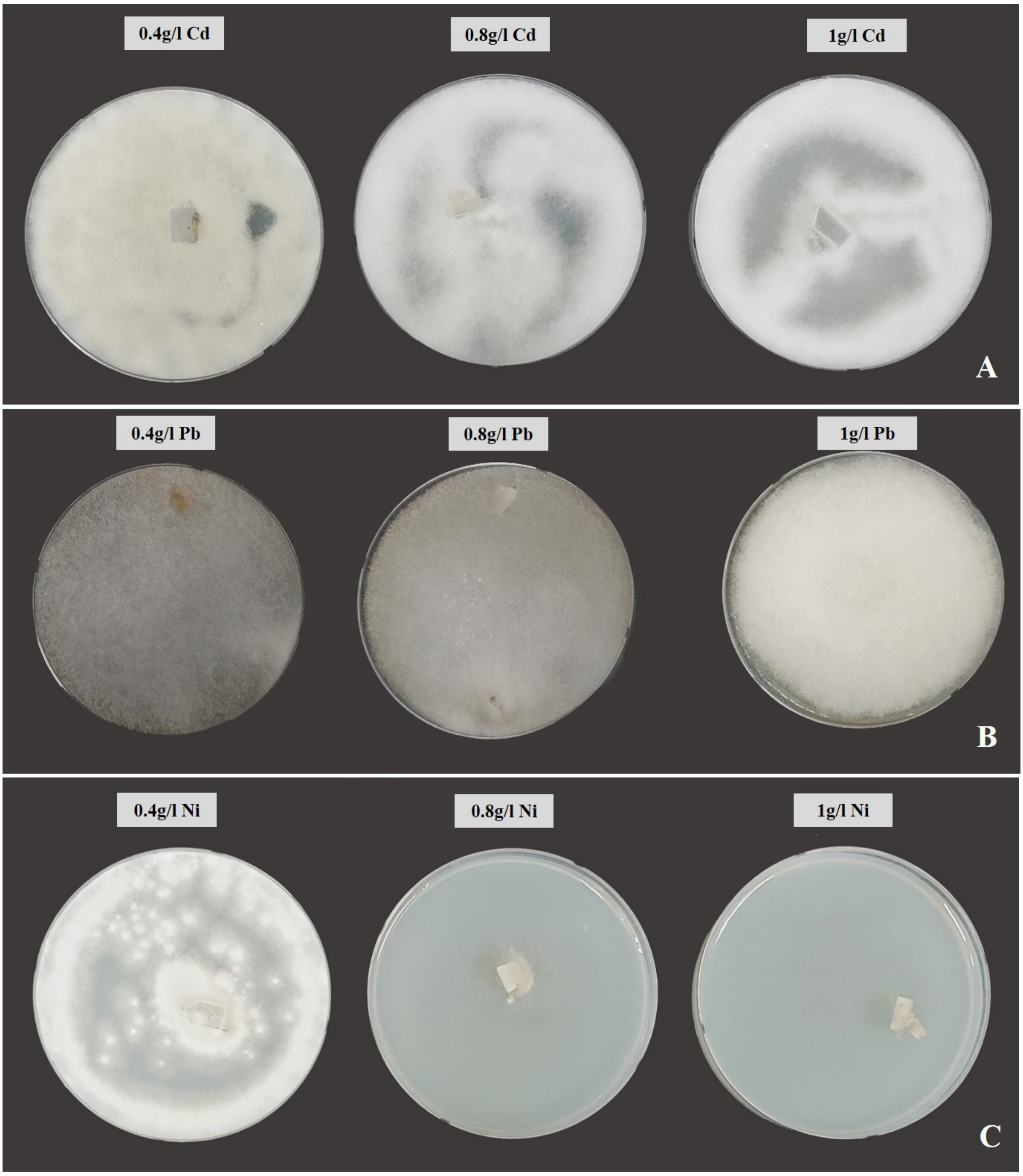


**Figure S3.** Heavy metal (Cadmium; Cd, Lead; Pb, Nickel; Ni) tolerance ability of the selected fungal strain (*Cunninghamella bertholletiae*) associated with *Solanum lycopersicum* in this study (**A**–**C**).

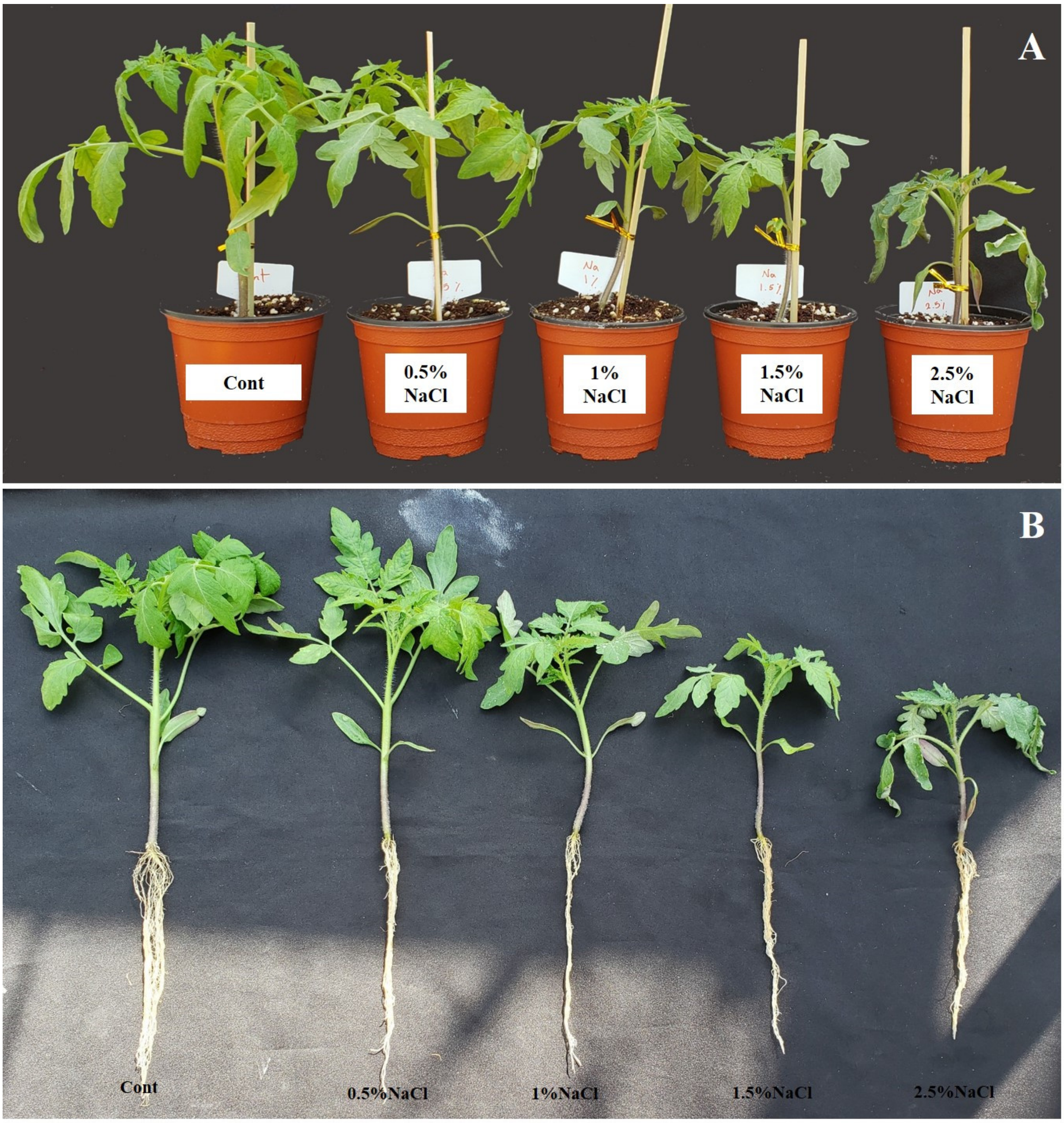


**Figure S4.** Effects of various sodium chloride (NaCl) concentrations on the growth of tomato seedlings following 10 days of treatment (**A** and **B**). Treatments: Cont (control), 0.5% NaCl, 1% NaCl, 1.5% NaCl, and 2.5% NaCl.

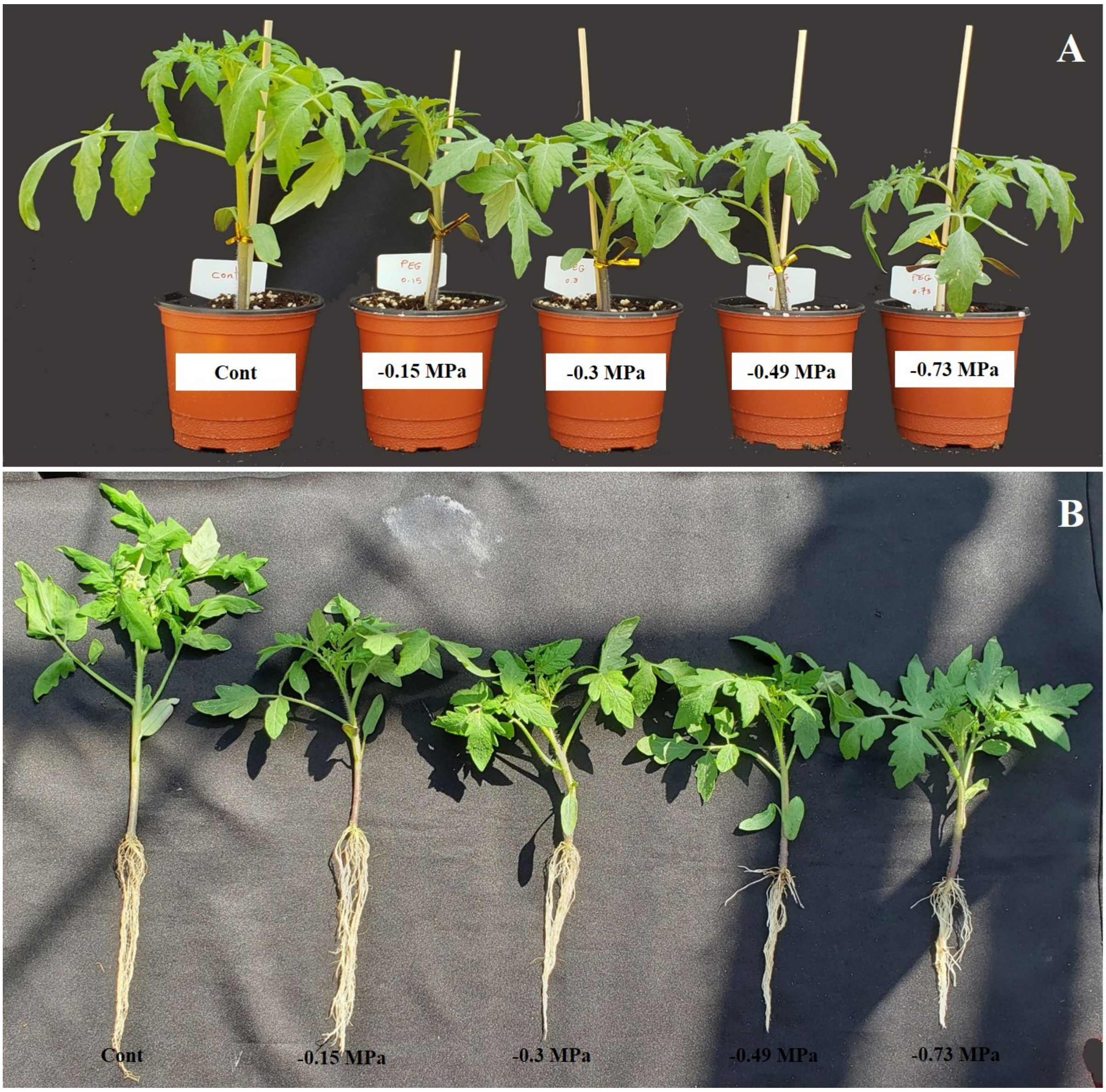


**Figure S5.** Effects of various polyethylene glycol (PEG 6000 Da) concentrations on the growth of tomato seedlings following 10 days of treatment (**A** and **B**). Treatments: Cont (control), PEG 5%; -0.15 MPa), PEG (10%; -0.3 MPa), PEG (15%; -0.49 MPa), and PEG (25%; -0.73 MPa).

**Table S1.** Effects of various sodium chloride (NaCl) concentrations on the growth of tomato seedlings. Data were calculated after 10 days of treatment.

| **Treatment** | **Plant height (cm)** | **Root length (cm)** | **Stem diameter (cm)** | **Leaf length (cm)** | **Leaf width (cm)** | **Chl (Spad)** | **Plant fresh weight (g)** | **Plant dry weight (g)** | **Root fresh weight (g)** | **Root dry weight (g)** | **No. leaf** |
|---|---|---|---|---|---|---|---|---|---|---|---|
| Cont | 18.1±0.64a | 24.0±0.0a | 0.55±0.02a | 16.28±0.53a | 12.56±0.70a | 31.76±0.52a | 10.21±0.90a | 0.63±0.03a | 1.17±0.45a | 0.09±0.0a | 8.6±0.24a |
| NaCl (0.5%) | 15.0±0.0b | 19.5±0.0b | 0.45±0.02b | 13.5±0.5b | 9.7±0.71b | 30.03±1.28b | 8.39±0.0b | 0.48±0.0b | 0.9±0.0b | 0.07±0.0b | 7.2±0.2b |
| NaCl (1%) | 13.8±0.37c | 18.0±0.0c | 0.40±0.01c | 11.9±0.18c | 8.3±0.46c | 28.64±1.33c | 5.52±0.0c | 0.36±0.0c | 0.82±0.05c | 0.06±0.0bc | 7.2±0.2b |
| NaCl (1.5%) | 11.7±0.12d | 16.0±0.0d | 0.34±0.01d | 10.6±0.53d | 7.7±0.7d | 26.9±0.80d | 4.36±0.0d | 0.32±0.0d | 0.51±0.04d | 0.05±0.0cd | 6.8±0.2c |
| NaCl (2.5%) | 10.0±0.54e | 14.0±0.0e | 0.30±0.01e | 9.1±0.18e | 7.1±0.55e | 25.3±2.45e | 3.22±0.0e | 0.26±0.0e | 0.49±0.0d | 0.04±0.0d | 6.2±0.2d |

Treatments: Cont (control), 0.5% NaCl, 1% NaCl, 1.5% NaCl, and 2.5% NaCl. Values show the means ± SD (n = 5) and significant differences at $p < 0.05$ (Tukey test). Data within the same column followed by different lowercase letters are significantly different.

**Table S2.** Effects of various polyethylene glycol (PEG 6000 Da) concentrations on the growth of tomato seedlings. Data were calculated after 10 days of treatment.

| Treatment | Plant height (cm) | Root length (cm) | Stem diameter (cm) | Leaf length (cm) | Leaf width (cm) | Chl (Spad) | Plant fresh weight (g) | Plant dry weight (g) | Root fresh weight (g) | Root dry weight (g) | No. leaf |
|---|---|---|---|---|---|---|---|---|---|---|---|
| 10DAT | | | | | | | | | | | |
| Cont | 18.1±0.64a | 24.0±0.0a | 0.55±0.02a | 16.28±0.53 a | 12.56±0.70a | 31.76±0.52a | 10.21±0.90a | 0.63±0.03a | 1.17±0.45a | 0.09±0.0a | 8.6±0.24a |
| PEG (-0.15) | 12.2±0.48b | 19.0±0.0b | 0.44±0.0b | 12.5±0.22b | 9.3±0.64b | 29.7±1.68b | 7.05±0.0b | 0.45±0.0b | 1.13±0.0b | 0.07±0.0b | 7.2±0.2b |
| PEG (-0.30) | 12.1±0.55c | 14.5±0.0c | 0.38±0.01c | 11.7±0.12b c | 9.1±0.18b | 28.66±0.57b | 7.0±0.0b | 0.43±0.0b | 1.04±0.06c | 0.06±0.0c | 7.0±0.0b |
| PEG (-0.49) | 11.8±0.48 d | 12.5±0.0 d | 0.36±0.01c d | 11.4±0.36c | 8.5±0.22c | 25.32±1.68c | 6.14±0.0c | 0.41±0.0bc | 0.93±0.05d | 0.05±0.0d | 6.8±0.2c |
| PEG (-0.73) | 10.6±0.53e | 11.0±0.0e | 0.34±0.02d | 10.6±0.4d | 8.0±0.31d | 21.7±1.38d | 5.8±0.0d | 0.38±0.01c | 0.91±0.0e | 0.04±0.0e | 6.4±0.24d |

Treatments: Cont (control), PEG 5%; -0.15 MPa), PEG (10%; -0.3 MPa), PEG (15%; -0.49 MPa), and PEG (25%; -0.73 MPa). Values show the means ± SD (n = 5) and significant differences at $p < 0.05$ (Tukey test). Data within the same column followed by different lowercase letters are significantly different.

**Table S3.** Physiochemical properties of soil samples during 10 days of treatment.

| Sample name | Soil texture | pH | EC (mS) | Moisture (%) |
|---|---|---|---|---|
| Cont | sandy loam | 8.0±0.0ab | 0.03±0.0f | 83.0±0.1e |
| PGPF | sandy loam | 7.95±0.05ab | 0.07±0.01f | 93.0±0.1c |
| S | sandy loam | 6.7±0.01c | 12.0±0.09a | 100.0±0.0a |
| S+PGPF | sandy loam | 7.7±0.0b | 5.35±0.25b | 96.0±0.9b |
| Dr | sandy loam | 7.7±0.1b | 0.63±0.05de | 45.0±0.0f |
| Dr+PGPF | sandy loam | 8.0±0.0a | 0.04±0.0f | 85.0±0.1d |
| Cd | sandy loam | 5.5±0.05d | 1.08±0.02cd | 100.0±0.0a |
| Cd+PGPF | sandy loam | 8.1±0.1a | 0.15±0.01ef | 96.0±0.18b |
| Pb | sandy loam | 5.0±0.15d | 1.25±0.01c | 100.0±0.0a |
| Pb+PGPF | sandy loam | 8.0±0.05ab | 0.06±0.01f | 98.0±0.2ab |

Treatments: Cont (control), PGPF (*Cunninghamella bertholletiae*), S (1.5% sodium chloride), S (1.5% sodium chloride) + PGPF (*Cunninghamella bertholletiae*), Dr (25% polyethylene glycol), Dr (25% polyethylene glycol) + PGPF (*Cunninghamella bertholletiae*), Cd (3mM cadmium), Cd (3mM cadmium) + PGPF (*Cunninghamella bertholletiae*), Pb (3mM lead), and Pb (3mM lead) + PGPF (*Cunninghamella bertholletiae*). Values are shown as the means ± SD (n = 5) and significant differences at $p < 0.05$ (Tukey test). Data within the same column followed by different lowercase letters are significantly different.

**Table S4.** Primers used for relative gene expression analysis.

| Gene symbol | Primers (5′–3′) Forward/reverse |
|---|---|
| SlACCase | 5′-CGCGATGAGGTTAGGTAAAAGGCA-3′<br>5′-GTCGATTCCCTTAAAAGTGGACGCA-3′ |
| SlAOS | 5′-CCGGCGGGAAGATCACGATG-3′<br>5′-TCGAAAACGGCGTCGTGTGA-3′ |
| SlICS | 5′-GGCAATAGATGCACTTCAGGCCA-3′<br>5′-CGCATGGTCCCAAGACGCTTT-3′ |
| SlRBOHD | 5′-TCAGGTCAAGCATCAAAGCCGTT-3′<br>5′-TGGTGAAACCGCAGCACAGT-3′ |
| SlGRAS6 | 5′-AAACCAAAGAGACCAGCTTCTGCG-3′<br>5′-AATAAAGTGCACCTGCCTCCTCCT-3′ |
| SlTAF1 | 5′-CACCATGACAGCAGCTGAATTGC-3′<br>5′-TCAAAAAGGCTTGTGCAGGTGCA-3′ |
| SlZH13 | 5′-ATGCCACTGATGGCTGTGGA-3′<br>5′-TGCGGTGGAAGTTACGGTGA-3′ |
| SlRING1 | 5′-TTGTGAGATGTCAGCACGAA-3′<br>5′-TCACCGAAGAAGAACAGCAC-3′ |
| SlCDF3 | 5′-GCTTGAGGGTCTTCCTCAGTTGA-3′<br>5′-AAAATCCTAAGACTCCATCAGAATCAG-3′ |
| SlActin | 5′-GGGATGGAGAAGTTTGGTGGTGG-3′<br>5′-CTTCGACCAAGGGATGGTGTAGC-3′ |
